# Strategies for tackling fake news in healthcare: a complex approach from a nursing perspective*

**DOI:** 10.1590/1980-220X-REEUSP-2025-0111en

**Published:** 2026-07-06

**Authors:** Luana dos Santos Costa, Carla Arena Aparecida Ventura, Sonia Silva Marcon, Sabrina da Costa Machado Duarte, Thiago Privado da Silva, Ana Larissa Araujo Nogueira, Ítalo Rodolfo Silva

**Affiliations:** 1 Universidade Federal do Rio de Janeiro, Escola de Enfermagem Anna Nery, Rio de Janeiro, RJ, Brazil.; 2 Universidade de São Paulo, Escola de Enfermagem de Ribeirão Preto, Ribeirão Preto, SP, Brazil.; 3 Universidade Estadual de Maringá, Maringá, PR, Brazil.

**Keywords:** Nursing, Health, Disinformation, Nurse Practitioners, Knowledge Management

## Abstract

**Objective:**

To unveil strategies for tackling fake news in the context of health and nursing from nursing professionals’ perspective.

**Method:**

This qualitative research, whose theoretical and methodological frameworks were, respectively, Complexity Theory and Grounded Theory, involved 25 nursing professionals from a public hospital and a Family Clinic in the city of Rio de Janeiro. Semi-structured interviews were conducted between 2021 and 2023.

**Results:**

The meanings revealed by nursing professionals considered the complexity of tackling fake news, highlighting that the dissemination of this information occurs through multiple channels and is embedded in social, political, and technological dynamics. The results indicated the importance of developing institutional fact-checking mechanisms and promoting educational campaigns as strategies to minimize the impacts of fake news on healthcare.

**Conclusion:**

The study’s complex perspective indicates that tackling fake news in health and nursing requires multidimensional approaches that combine education, regulation, and the valuing of science in the construction of reliable knowledge.

## INTRODUCTION

From a postmodern perspective, the ontological understanding of being prioritizes a multifaceted reality, conceived as dynamic and in constant transformation. In this regard, language plays a central role in the construction of reality so that any claim to achieve objective truth is configured as a meta-discourse^(1,2)^. Thus, new narrative and methodological forms emerge, consistent with the challenges of the new times, in which fake news, despite being mechanisms usually used in political debates, also begin to affect the narratives that permeate the context of public health^(3)^.

In recent years, there has been an expansion of fake news in anti-vaccine movements^(4)^, which has impacted the return of diseases considered, until then, to have been eradicated. These are publications on social networks (posts), audios and videos that go viral on the internet and validate the misleading perception that the vaccine is dispensable. An important milestone in this process, according to the World Health Organization, was the COVID-19 pandemic, which highlighted fake news as a global phenomenon of importance to public health^(5)^.

Currently, the dynamics of access to and dissemination of information aimed at building and using knowledge are influenced by Information and Communication Technologies (ICTs). ICTs also establish new and defining forms of knowledge standards and behaviors that affect the work process and healthcare services, as they impact the actions of people who access these services^(6)^.

The nursing work process is related to the transience, discontinuity, and chaos of postmodern times. Thus, new organizational principles have emerged that also imply the need for the nursing team to perform multiple functions in a decentralized manner, with flexibility, leadership, risk-taking, effective communication, relational skills, among others^(7)^. In this context, ICTs influence the nursing knowledge management process, from access to and dissemination of information among the team to decision-making in direct patient care^(6)^. Within the dynamic nature of this complex phenomenon lies the reality of fake news as a global problem affecting all sectors of society, and nursing is no exception.

Given the above, it is understood that the nursing work process can be affected by fake news, due to its influence on the elements that involve these professionals’ work reality, such as object, agent, instrument, purpose, method, and product, causing modifications and possible harm in all dimensions of their work. In this context, fake news should be considered a challenge for the development of effective health guidance strategies for the population and for knowledge management in the work context, as well as for nurses’ political competency^(8)^.

Thus, it is assumed that the meanings attributed by nurses to fake news can guide their conduct in addressing this important public health problem and, in fact, influence the nursing work process through the management of knowledge involved in the dynamics of access, dissemination, and consumption of verified information, for the use of relevant scientific bases in the context in which they provide care, especially for guiding patients who may consume and disseminate fake news.

From a systemic perspective, this research considers the paradigm of complexity by recognizing that knowledge is a relational process, in which Complexity Theory considers that the process of knowing results from the articulation between the neurobiological dimensions of the brain and the sociocultural elements that shape society^(1)^. Therefore, the phenomenon of fake news in health and nursing cannot be understood without considering nursing professionals’ perspectives.

Given the above, this study aims to unveil strategies for tackling fake news in the context of health and nursing from nursing professionals’ perspective.

## METHOD

### Study Design

This is qualitative research, whose theoretical and methodological frameworks were, respectively, Grounded Theory (GT)^(9,10)^ and Complexity Theory^(1)^. For the research’s analytical reliability, the Equator guidelines were used, notably the COnsolidated criteria for REporting Qualitative research guide.

### Selection Criteria

The study included nurses and nursing technicians, divided into two sample groups: 1) Primary Health Care professionals (Family Clinic); 2) Tertiary Health Care professionals (hospital). Participants with at least one year of professional experience in the institution and in direct patient care as a nurse or nursing technician were included. In hospital settings, data were collected from professionals working in adult inpatient units (medical and surgical clinics). Participants on leave of absence for any reason were excluded. Participants were invited in person, without prior contact, at the study setting, using convenience sampling. There were no refusals or withdrawals from any participant.

### Study Site

Data were collected in two settings (a federal university hospital and a Family Clinic), both located in the city of Rio de Janeiro. The hospital setting has several clinical specialties and approximately 550 beds. The chosen Family Clinic was inaugurated in December 2010, a unit with 14 Family Health Strategy teams serving approximately 52,000 people. The choice of two settings was based on the hypothesis that the context can influence the dynamics of access to and dissemination of information, both in terms of nursing professionals and the patient profile in each sphere of healthcare.

### Population

Nurses and nursing technicians working in the Brazilian Unified Health System, where the first sample group consisted of Primary Health Care professionals working in Family Clinics, and the second sample group consisted of Tertiary Health Care professionals working in hospital settings.

### Data Collection

For participant characterization, a form with sociodemographic questions was used. For the analytical corpus, semi-structured, in-person, individual interviews (principal researcher and interviewee) were employed, recorded on an electronic device. The guiding questions were developed after bibliographic research and due to the thematic depth. The guiding questions were: what do you understand by fake news? How do you perceive this phenomenon in the context of health and nursing? What can be done, within the scope of nursing, to address fake news in healthcare? Circular questions were used as participants presented answers that indicated the need for further exploration. Participants were instructed to answer the questions at their own pace, with the possibility of interrupting the interview if they deemed it necessary. As data collection occurred in parallel with the analytical process, a characteristic of the Theory of Motion, a pilot test was not conducted.

Data were collected between November 2021 and October 2023, at the research settings, on dates and times indicated by participants, and in a private setting. Data collection was carried out by the principal investigator (LSC), who is a nurse with a master’s degree and, at the time, a doctoral candidate in nursing, with no affiliation to the research setting and no conflicts of interest with the institution or the professionals. The principal investigator is competent in data collection techniques and methodological framework, based on training with the supervising researcher (IRS) as well as in previous research.

The interviews lasted an average of 30 minutes. Considering the nature of the method, in which the meanings presented by research participants must emerge from inductive and deductive processes^(10)^ during the interview, especially from circular questions, the interviews were not returned to participants for additions or adjustments.

Data collection was interrupted upon reaching theoretical saturation, from the GT authors’ perspective, through a process of simultaneous data collection and analysis. Thus, in the analytical course, the researchers discussed the process of identifying concepts/categories and subcategories with sufficient theoretical density to allow for an understanding of the investigated reality^(9,10)^. This process is facilitated by the use of memos (reflective field notes), analytical resources employed during and after the interviews^(9)^.

The memos allow for the direction of potential new questions in subsequent interviews, so there was no need to repeat interviews^(9)^. Furthermore, these resources allowed for reflections on the development of concepts, thus aiding in the understanding and consensus regarding theoretical data saturation, which was duly discussed among the researchers.

### Data Analysis

Data were analyzed by all authors, who were doctoral and doctoral students in nursing, as well as university professors. The interviews were subjected to analysis following the coding stages of GT from a Corbinian perspective^(9)^, which are open, axial, and integration. In open coding, data were segmented into distinct parts and meticulously examined, the comparison of which aimed to capture similarities between the initial data (preliminary codes). From the comparative analysis of the preliminary codes, these were grouped, which allowed the delimitation of conceptual codes, presented as abstract representations of facts, objects, or actions perceived as significant by the researchers. The grouping of conceptual codes by similarities gave rise to subcategories and, through analytical density and connections between them, the category was defined^(9,10)^.

The subcategories were ordered based on the paradigmatic model, which considered three components: 1) conditions, which deal with the factors that influence the development of the investigated phenomenon; 2) actions-interactions, which deal with the strategies for developing and addressing the problem; 3) consequences, which indicate the potential reactions resulting from the implemented strategies^(9)^.

### Ethical Aspects

This research was approved by the Research Ethics Committee, under Opinions 4,970,482, 5,036,468, and 5,118,815. Participants were informed about the research’s nature and purpose, as well as the researchers’ motivations, and signed the Informed Consent Form. Their identities were kept anonymous, as they were characterized throughout the article as follows: HN (hospital nurse); HNT (hospital nursing technician); PCN (Primary Care nurse); and PCNT (Primary Care nursing technician). The acronyms followed by the number of their respective interviews were used.

## RESULTS

Twenty-five nursing professionals participated in the study, of whom 17 were registered nurses (11 from the hospital and six from the Family Clinic) and eight were nursing technicians (six from the hospital setting and two from the Family Clinic). The mean age of the professionals working in the hospital was 35 years. In the Family Clinic setting, the mean age was 28 years.

The mean length of service for nursing professionals in the hospital was 16 years for nursing technicians and 11 years for nurses. For professionals in the Family Clinic, the mean was two years for nursing technicians and four years for nurses. All nurses held at least a graduate degree. Only three were nursing technicians.

From the analytical process emerged the concept/category “Strategies for tackling fake news in healthcare: a complex approach from a nursing perspective”, which, using the paradigmatic model, is supported by the following subcategories, distributed as follows in the paradigmatic model of the method: “Fake news operators: who are they and how to avoid them? (conditions)”; “Reliable sources: arguments from authority or authority of arguments?”; “Knowing in order to intervene: elements for identifying fake news (actions-interactions)”; “Ways to tackle fake news (consequences)”. It is important to highlight that the excerpts from statements used throughout the article are merely illustrative of the contextualization of results, as the raw data (interviews) are more extensive than their fragments.

### Fake News Operators: Who are they and how to Avoid them?

This subcategory, from nursing professionals’ perspective, identifies the most significant operators of fake news (those who create, disseminate, and/or consume false news), who are divided into two groups. The first group consists of people or sources of information who are close to patients and with whom there is interaction, such as the patients themselves, caregivers, family members, and neighbors.

Nursing professionals considered that interactions between fake news spreaders and patients, when relationships of trust are established, imply interference in professional practice and, consequently, in nursing care. For nurses and nursing technicians, the influence of informal, but reliable, sources from patients’ point of view results in distortions of health practices and quality of care, a scenario perceived as worrying by the healthcare professionals investigated, especially because it weakens the authority of their arguments, thus implying the devaluation of safe health and care practices.

*They listen to their neighbors, to the website Google, and disproving them is no easy task.* (HN11)

*We notice this in our daily lives with patients and our families. I don’t think there’s anyone today who doesn’t know a family member who posts fake news daily in groups.* (PCNT16)

*And patients are completely ignorant when it comes to health matters. So, when they receive* (fake news) *from neighbors or a relative, they end up believing and spreading this misinformation.* (HN4)

*Often, this information about our care is distorted, lacking any proven evidence, and comes from a family member or friend, making this fake news seem safer for the patient. This ends up influencing them because, since they don’t have a family relationship with them, everything we say is questioned.* (PCN13)

*Because a patient comes with information that they believe is correct, because someone they know said so. Often, it’s not even a healthcare professional. They said it, and they* (patient) *think it’s right.* (HN3)

*Some believe it, but others don’t. They do it because their mother said so, their grandmother said so* [...] *you’re going to administer the prescribed medication, then a patient comes along refusing to take it because it’s harmful, because they saw on Google that it affects some system, or because someone said so.* (HN7)

The second group identified by nursing professionals consisted of individuals distant from the personal lives of patients and the professionals themselves, but who, similarly to the previous group, exert considerable influence on the process of understanding reality, as well as on the construction of meanings about health among those who consume and disseminate fake news. In this context, they identified political leaders, artists, and digital influencers as members of this group.

It is also worth highlighting participants’ perception of health information politicization, the focus of which may be related to the interests of those who produce and disseminate fake news, potentially compromising trust in health institutions and professionals.

*During the COVID-19 pandemic, there were posts and messages from leaders talking about subjects they knew absolutely nothing about.* (HNT3)

*For instance, lately, regarding vaccines, some people are spreading information that contradicts their effectiveness, discouraging the population from getting vaccinated. It’s very much intertwined with politics as well, I think, and because of that, it harms health policies.* (HN6)

*And there’s also a political issue, for instance, the government spreading false information for its own benefit.* (HN8)

*We receive a patient who says they saw a video on YouTube* ^®^ *from a certain person. Often, the person giving that information isn’t even a professional.* (PCN18)

*Unfortunately, we have a population that is not very educated, that doesn’t understand the health-disease process, and believes everything they see on the internet and ends up spreading it. They let themselves be swayed by influencers who appear in the media. For instance, artists, politicians whom they admire. When people in this position end up saying something they don’t know, they have the role of influencing laypeople.* (HNT3)

Nursing professionals recognize that those who spread fake news establish such interactions significantly in the virtual setting through social media. The meanings attributed by participants to these platforms are related to the dangers they pose, especially due to the dynamics they have in the processes of disseminating and accessing fake news.

*The internet, Instagram* ^®^ *, which is surreal, is very dangerous, and WhatsApp* ^®^ *, which is even more surreal.* (HN24)

*Especially social media. Instagram* ^®^ *, Twitter* ^®^ (“X”)*, I think they spread a lot of fake news.* (HN23)

*Lately, fake news has gained traction thanks to WhatsApp* ^®^ *, right?!* (PCNT20);

*Fake news is being spread* [...] *through social media.* (HNT12)

### Reliable Sources: Arguments from Authority or Authority of Arguments?

Nursing professionals assign meanings that signal reliability to official sources from established institutions, such as the Ministry of Health, Municipal Health Departments, and the Technical Standards originating from these contexts. They recognize the reliability of journalistic sources, especially those that corroborate governmental sources.

The recognition of reliable sources by nursing professionals is centered on the argument of authority that such sources represent. In this regard, they value specialized organizations such as the *Fundação Oswaldo Cruz* and the Brazilian National Health Regulatory Agency. Furthermore, participants highlighted the importance of easy access to information through social media used by governmental and specialized agencies.

*I believe you should seek information from the Technical Standards, which are published by the Health Department, pay attention to the city’s social media channels, and participate in the training sessions that are offered.* (PCN17)

*I think that nowadays, everything is too easy. Even the Ministry of Health and Anvisa* (Brazilian National Health Regulatory Agency) *have Instagram* ^®^ *accounts to debunk misinformation on social media. So, these organizations have entered social media to educate. I try to do that through these organizations.* (HN6)

*The Ministry of Health, for me, is a reliable source. Sometimes, when it’s a health news story that’s been in the newspapers, they say, you know?! Look for the most reliable journalistic source.* (HN11)

*I think it’s the government agencies, the Ministry of Health, Fiocruz. In my opinion, they have a group of experts who will study the situation and be able to implement the correct measures.* (PCNT20)

From a decentralized perspective, nursing professionals attributed reliability to sources established in daily work interactions, such as nursing supervisors/leaders and other nurses. However, they also revealed a certain degree of skepticism regarding seeking information from professionals who, although recognized by them as knowledge references, were not always unequivocally perceived as reliable sources of information.

*We try to find a way. When something is directly related to the hospital, we ask the management. At least, I do.* (HNT5)

*I consider the nurse’s knowledge to be an important source.* (PCNT16)

*So, I think they should be suspicious at first, and then always listen to people who are experts in that field, those who study the subject. Nurses can be reliable sources.* (HN2)

Participants also listed traditional media outlets, such as radio, television, and newspapers, as reliable sources of information. Their trustworthiness stems from the authority of the arguments that nurses and nursing technicians understand, a belief established through the commitment and investigative capacity of these media outlets.

*By searching through reliable news sources, such as newspapers that already have a process for verifying information.* (HN25)

*More traditional sources of information, such as newspapers, even online ones, that include the name and reference of the source of information.* (HN14)

*On the internet, you post whatever you want. Journalism on television, however, needs a solid foundation to convey that information to you. Besides reporting the news, they go to the location, they ask questions. There’s an investigative process involved.* (HN26)

*I always look for information in databases and also on television news programs, because they already have mechanisms for fact-checking. After the COVID-19 pandemic, I think this has intensified even more.* (PCN19)

For the nurses interviewed, reliable sources of information are those directly related to science, from specialized databases such as LILACS, BDENF, PubMed, as well as more generic tools like Google^®^. In this context, scientific journals are considered reliable sources for specific approaches, such as case discussions.

*The actual health databases. LILACS, BDENF, PubMed are the ones that give us the most support, not Google* ^®^ *.* (PCN14)

*I believe that nurses must rely on information from reliable sources, such as databases, online libraries, and scientific journals.* (HN04)

*Search for articles in databases such as LILACS and BDENF, which contain collections of scientific journals.* (HN06)

*We also have access to the articles, so whenever there is a discussion of cases, we search the scientific journals and databases.* (PCN17)

### Knowing in Order to Intervene: Elements for Identifying Fake News

According to study participants, fake news presents structural clues that allow one to identify its false nature. Therefore, some actions were suggested, such as the need to verify the source of information and compare it with other sources considered reliable.

As explained above, for nursing professionals, fake news can present, within its overall information, a true component that is associated with a false one. Hence, they mention authorship, the communication channel through which the news is disseminated, and the method used as indicators that guarantee the reliability of the information accessed and disseminated.

*These news stories are like a game of telephone. They contain a tiny grain of truth, but whoever keeps forwarding them always adds lies.* (HN8)

*Sometimes, something is mentioned, and this happens a lot in the health field. People see that something is being studied, and they immediately mix in data from other studies, distorting the information.* (HN22)

*So, check who the author of that material is, look for information in more than one source. If it’s a video, check the video quality, whether it has been edited.* (HN23)

*Believing that research involves observing who wrote that information. Checking if they are a professional who truly knows about that subject.* (HNT21)

*Furthermore, be very careful when reading this false information; it usually comes with a sensationalist title and a well-known name that always leaves us in doubt.* (PCN15)

*I think they should, at first, be suspicious. Then, they should always listen to people who are experts in that field, those who study the subject.* (HN2)

However, despite identifying these identifying elements, the respondents considered fake news to be difficult to recognize, as demonstrated in the following excerpts:

*When we see the news on social media, it becomes difficult to distinguish what is false or true in that information, because, with each passing day, it becomes more elaborate.* (HN1)

*Sometimes we get confused when we come across this false information. Because, nowadays, the resources are also very advanced.* (PCNT20)

*I’m not going to say it’s an easy task. It’s not always possible to identify it at first glance.* (PCN19)

### Ways to Tackle Fake News

This subcategory encompasses the data presented by study participants regarding actions that, according to them, result in tackling the spread of fake news in healthcare, even within the context of nursing work, especially with patients. To this end, the data revealed the importance of health education as the main strategy used by professionals.

The resources mentioned by the interviewees present different possibilities for tackling fake news, especially those focused on guidance and visual resources, such as the creation of booklets addressing health issues.

*I think the only way is to educate patients and their companions. That’s what we’ve been doing here, bringing in someone who works in that area to explain things.* (HNT07)

*I think informational booklets could be an option. I believe that this education about misinformation is in everyone’s interest.* (HN2)

*Daily guidance for patients and their caregivers. And I think it’s important that these educational actions involve the entire community, health workers, community representatives, and schools.* (PCNT16)

*I usually talk to and advise patients or family members who believe false news spread on social media. They don’t always listen, but I do my job of guiding them.* (HN1)

*I think health education, which is something that is very present, including in Primary Care, is also crucial. I think that talking to and informing people, bringing knowledge to people, is a fundamental tool, basically for tackling fake news.* (PCN15)

Another key educational resource highlighted is the need for awareness campaigns aimed at tackling the myths and taboos that contribute to the spread and repetition of fake news.

*I think there should be awareness campaigns regarding the sharing of this information, because this information ends up having this magnitude because there are people who share it.* (HN4)

*I think campaigns within the hospital are important to raise patient awareness regarding certain care practices. I believe that, whenever possible, there should be educational initiatives: these could be lectures, discussion groups* [...] *I think we should get closer to the patient, trying to understand their doubts and clarify them point by point, as I do when I encounter situations where there is a lack of information.* (HNT3)

*One interesting action that I think could be taken, similar to what’s done with vaccination, is awareness campaigns, because they can sensitize and alert people that fake news causes problems.* (PCN14)

Hospital nurses mentioned, as a strategy to tackle fake news, the development and promotion of fact-checking profiles on social media, with Workplace^®^ being a particular highlight.

*I think there should be a reliable source, a platform, I don’t know, where you can replicate the information and, in the end, it can tell you if it’s true or not.* (HN6)

*I see that something like Workplace* ^®^ *could be created for the public. It’s similar to Facebook* ^®^ *, but with the aim of disseminating secure information and actions within the institution. I think it’s an interesting idea that could be used as a strategy, as it would be like a social network for the hospital, and professionals could manage the information there.* (HN08)

Furthermore, participants highlighted continuing education as an important strategy for nursing professionals, through courses or lectures, with the aim of empowering them with knowledge and attitudes, in order to transfer this knowledge to patients.

*I think we professionals need training courses, courses or lectures that clarify doubts about the subjects that are usually pointed out in fake news.* (HN4)

*These training sessions are important because they give us confidence. They make us reflect on our daily work; they bring about new exchanges. This makes us feel more prepared to deal with this news when the user brings it to us.* (PCN17)

Therefore, from the set of principles/subcategories, emerged the substantive theory, based on complexity, which signals the multidimensionality for tackling fake news in healthcare, from a nursing perspective. The objective is to recognize the connections between the operators of fake news, the sources of information and their symbolic implications, between arguments of authority and the authorities of the argument, as well as the multiple ways to tackle fake news in healthcare.

## DISCUSSION

The context from which fake news emerges is as influential, or even more so, than the false information itself. In this sense, political and health leaders must commit to public policies and mobilizing strategies for knowledge capable of breaking the logic based on post-truth, which is limited to the realm of emotions^(8,11)^. This reality is reinforced by virtual bubbles, driven by communication technologies such as WhatsApp^®^, Facebook^®^, Instagram^®^, “X”^®^, and many others. Together, these media encourage interactions restricted to the perspectives common to virtual affiliation groups, thus reiterating the non-dialectical process of knowledge within restricted groups^(4,11,12)^.

From a complexity perspective^(1)^, the interdependence between a group and its members is not established solely by the holographic principle, in which the whole contains the parts and the parts represent the whole. For complex thought, the dynamics of interaction between these dimensions give rise, above all, to the principle of the recursive circuit, whose actors are both producers and products of themselves. Thus, the reiteration of common thought that reifies fake news is based on the quality of the interactions between the parts and the whole, whose relationship of trust exerts reciprocity between them, becoming a preponderant factor in the motivating actions for the consumption and sharing of fake news^(13)^.

The interaction between concepts and principles in this research supports the recursive circuit principle by establishing a producer-product effect on the emitters of fake news, since, while they emit false news, they are also consumers dependent on it^(1)^. In this cyclical process, the scientific literature reiterates that, in the context of health, when dealing with fake news, the discourse of authority prevails over the authority of discourse^(14)^, which is why health and research institutions, as well as nurses themselves, must establish strategies that allow them to achieve projections that value science^(15)^. Therefore, these actors must be understood as epistemic authorities, facilitators of access to information for knowledge construction^(16,17)^, which aims, among other objectives, to confront fake news in science and health^(18)^.

This research supports what has been discussed in the scientific literature regarding the magnitude of the influence of actors (leaders and institutions) that shape the perspective of the authority of discourse on the dissemination and/or tackling of fake news. However, the level of scientific knowledge of people who consume and share fake news is also an intervening factor in this reality^(19)^, thus reiterating the need for better investments in nurses’ scientific training^(20)^. In this context, it is understood that nurses, throughout their training, must be prepared to develop leadership skills, among which is the essential ability to mobilize efforts towards a common purpose, under a logic capable of motivating through example, which includes valuing science. Thus, it is the nurses’ role to remain informed and up-to-date on matters that may influence their decision-making processes, which involves the need for epistemic vigilance and scientific proficiency to interpret data and decode information that is assumed to be scientific^(21)^.

Given the above, it is possible to infer that tackling misinformation involves the very complexity of knowledge management, such as the ability to consume, decode, communicate, and assess scientific information aimed at generating knowledge, the consequences of which may reflect behaviors that affect health. Therefore, it is also necessary to know the dynamics and origin of the information consumed^(17)^, as well as the assumption of the need for technological and scientific proficiency for access, consumption, and dissemination of true information.

Several actions can contribute to vigilance in tackling misinformation caused by fake news in the health field. The data from this research also corroborate indications of epistemic surveillance of information, since, in journalism, for instance, guaranteeing the integrity of information is the responsibility of the journalistic context itself, whether by the professional, the media outlet, or associations related to the commitment to the veracity of news in the press. However, other measures can make a difference, such as verifying the date of the information disseminated^(22)^, since the temporal space can cause obsolete information to constitute fake news when disseminated as a set of current data. Furthermore, the speed at which fake news spreads is greater than the dynamics of strategies to tackle it, among which are journalistic fact-checking agencies, which operate according to user demand, signaling the need to verify these news items^(23)^.

The use of technological resources in health education initiatives is also an important strategy for tackling misinformation. However, it is crucial to emphasize the importance of nursing professionals seeking out these resources and providing coherent, high-quality, scientifically sound information^(24)^. Therefore, the process of communicating science, especially in the field of health, should be a skill that nursing professionals must constantly develop^(20)^.

Regarding the sources, the results of this research are supported by evidence indicating the nature of information sources as an element that integrates the multidimensionality of access to and dissemination of fake news in health, originating from social media. A study conducted by Iowa State University with university students demonstrated that the majority (69%) of participants were able to successfully determine the veracity of health-related publications on social media. However, it highlighted that, among the elements that guided students’ judgment, the credibility given to the source, the appearance, and the design style of the posts were primarily key factors^(19)^.

From a complex perspective, the spread of fake news in healthcare must be understood through a contextual analysis that highlights the nuances involving socioeconomic and cultural elements^(25)^. This understanding is supported by research conducted with 5,307 respondents in six sub-Saharan African countries, whose data highlighted that, although a significant portion of fake news about health is disseminated accidentally, a considerable number of individuals intentionally share such information. However, the most significant aspect of this data lies in individual determinants, which indicated that older men, more prone to risk-taking, are the actors most inclined to share false health information^(25)^.

The data from this research are therefore supported by the literature, particularly regarding the multidimensionality involved in the consumption and dissemination of fake news in the health field, based on the importance of context, sources, and the reiteration of news^(8,16,19,24)^. However, they stand out for presenting a complex perspective on nursing regarding the topic, especially since this is the profession that occupies the largest number of jobs in the health sector across all systems on the planet, as well as being strategic for tackling misinformation in the field^(8)^.

The research limitations are related to the epistemic field, since the nature of the participants, being solely within the public health context, may, for instance, lead to specific meanings regarding fake news in health and nursing.

## CONCLUSION

The meanings revealed by nursing professionals considered the complexity of tackling fake news in healthcare and in nursing itself, highlighting that the dissemination of this information occurs through multiple channels and is embedded in social, political, and technological dynamics. For nurses and nursing technicians, regardless of their work setting, there are significant challenges in dealing with patients influenced by unreliable sources, whether family members, social media, or public figures. Furthermore, the results of this research achieved the study’s objective by indicating the importance of developing institutional verification mechanisms and promoting educational campaigns as essential strategies to minimize the impacts of fake news on healthcare.

## DATA AVAILABILITY

The entire dataset supporting the results of this study was published in the article itself.
