# Supplementary material for: Strategies for tackling fake news in healthcare: a complex approach from a nursing perspective
Source: Rev Esc Enferm USP. 2026 Jul 6;60:e20250111. doi: 10.1590/1980-220X-REEUSP-2025-0111en (PMC13345668; doi:10.1590/1980-220X-REEUSP-2025-0111en)
Supplement: Translation

# Translation: Estratégias para o enfrentamento de fake news na saúde: uma abordagem complexa sob a perspectiva da enfermagem*

**DOI:** 10.1590/1980-220X-REEUSP-2025-0111pt

**Authors:** Luana dos Santos Costa, Carla Arena Aparecida Ventura, Sonia Silva Marcon, Sabrina da Costa Machado Duarte, Thiago Privado da Silva, Ana Larissa Araujo Nogueira, Ítalo Rodolfo Silva

**Affiliations:** 1 Universidade Federal do Rio de Janeiro, Escola de Enfermagem Anna Nery, Rio de Janeiro, RJ, Brasil.; 2 Universidade de São Paulo, Escola de Enfermagem de Ribeirão Preto, Ribeirão Preto, SP, Brasil.; 3 Universidade Estadual de Maringá, Maringá, PR, Brasil.

**Keywords:** Enfermagem, Saúde, Desinformação, Profissionais de Enfermagem, Gestão do Conhecimento

## Abstract

**Objetivo:**

Desvelar as estratégias para o enfrentamento de fake news no contexto da saúde e da enfermagem sob a perspectiva dos profissionais de enfermagem.

**Método:**

Pesquisa qualitativa, cujos referenciais teórico e metodológico foram, respectivamente, a Teoria da Complexidade e a Grounded Theory. Participaram 25 profissionais de enfermagem de um hospital público e de uma Clínica da Família da cidade do Rio de Janeiro. Foram empregadas entrevistas semiestruturadas entre 2021 e 2023.

**Resultados:**

Os significados desvelados pelos profissionais de enfermagem consideraram a complexidade do combate às fake news, evidenciando que a disseminação dessas informações ocorre por múltiplas vias e está inserida em dinâmicas sociais, políticas e tecnológicas. Os resultados sinalizaram a importância do desenvolvimento de mecanismos institucionais de checagem e promoção de campanhas educativas como estratégias para minimizar os impactos das fake news no cuidado em saúde.

**Conclusão:**

A perspectiva complexa do estudo sinaliza que o enfrentamento das fake news na saúde e na enfermagem exige abordagens multidimensionais que combinem educação, regulamentação e valorização da ciência na construção do conhecimento confiável.

## INTRODUÇÃO

Na perspectiva pós-moderna, a compreensão ontológica do ser privilegia uma realidade multifacetada, concebida como dinâmica e em constante transformação. Nesse sentido, a linguagem assume um papel central na construção da realidade, de modo que qualquer pretensão de alcançar a verdade objetiva se configura como um metadiscurso^(1,2)^. Assim, emergem novas formas narrativas e metodológicas, condizentes com os desafios dos novos tempos, nas quais as *fake news,* apesar de serem mecanismos usualmente utilizados nos embates políticos, passam a incidir também sobre as narrativas que permeiam o contexto da saúde pública^(3)^.

Nos últimos anos, observou-se a expansão das *fake news* nos movimentos antivacina^(4)^, o que impactou o retorno de doenças consideradas, até então, erradicadas. São publicações em redes sociais (*posts*), áudios e vídeos que viralizam na internet e validam a percepção enganosa de que a vacina é dispensável. Um marco importante nesse processo, de acordo com a Organização Mundial da Saúde, foi a pandemia de COVID-19, que evidenciou as *fake news* como fenômeno global de importância para a saúde pública^(5)^.

Atualmente, a dinâmica de acesso e a disseminação de informações que visam à construção e utilização de conhecimentos são influenciadas pelas Tecnologias de Informação e Comunicação (TICs). As TICs estabelecem, de igual modo, formas novas e definidoras de padrões e condutas de conhecimentos que afetam o processo de trabalho e serviços de saúde, uma vez que alcançam as ações das pessoas que acessam tais serviços^(6)^.

O processo de trabalho da enfermagem se relaciona à transitoriedade, à descontinuidade e ao caos dos tempos pós-modernos. Assim, têm-se configurado novos princípios organizacionais que implicam, também, a necessidade de a equipe de enfermagem desempenhar múltiplas funções, de forma descentralizada, com flexibilidade, liderança, assunção de riscos, comunicação efetiva, habilidades relacionais, entre outros^(7)^. Nessa conjuntura, as TICs influenciam o processo de gestão do conhecimento da enfermagem desde o acesso e disseminação de informações entre a equipe até a tomada de decisão no cuidado direto ao paciente^(6)^. Na dinamicidade desse fenômeno complexo, está a realidade das *fake news* como problema global que afeta todos os setores do tecido social, não sendo diferente para a enfermagem.

Diante do exposto, entende-se que o processo de trabalho da enfermagem pode ser afetado por *fake news*, devido às suas influências nos elementos que envolvem a realidade laboral desses profissionais, como objeto, agente, instrumento, finalidade, método e produto, acarretando modificações e possíveis prejuízos em todas as suas dimensões de trabalho. Nessa conjuntura, as *fake news* devem ser consideradas um desafio para a elaboração de estratégias efetivas de orientação em saúde para a população e para a gestão do conhecimento no contexto do trabalho, bem como competência política do enfermeiro^(8)^.

Desse modo, sustenta-se o pressuposto de que os significados atribuídos pelos enfermeiros às *fake news* podem direcionar suas condutas no enfrentamento desse importante problema de saúde pública e, de fato, influenciar o processo de trabalho da enfermagem a partir da gestão do conhecimento envolvido na dinâmica de acesso, disseminação e consumo de informações verificadas, para a utilização de bases científicas pertinentes no contexto em que exercem o cuidado, sobretudo para a orientação de pacientes que podem consumir e disseminar *fake news*.

Sob uma perspectiva sistêmica, esta pesquisa contempla o paradigma da complexidade por reconhecer que o conhecimento é um processo relacional, no qual a Teoria da Complexidade considera que o processo de conhecer resulta da articulação entre as dimensões neurobiológicas do cérebro e os elementos socioculturais que conformam a sociedade^(1)^. Assim, não se pode compreender o fenômeno das *fake news* na saúde e enfermagem sem, contudo, atentar para as perspectivas apresentadas pelos profissionais de enfermagem.

Diante do exposto, o estudo tem como objetivo desvelar as estratégias para o enfrentamento de *fake news* no contexto da saúde e da enfermagem sob a perspectiva dos profissionais de enfermagem.

## MÉTODO

### Tipo do Estudo

Pesquisa qualitativa, cujos referenciais teórico e metodológico foram, respectivamente, a *Grounded Theory* (GT)^(9,10)^ e a Teoria da Complexidade^(1)^. Para a confiabilidade analítica da pesquisa, foram empregadas as diretrizes do *Equator*, notadamente o guia *COnsolidated criteria for REporting Qualitative research*.

### Critérios de Seleção

Participaram do estudo enfermeiros e técnicos de enfermagem, distribuídos em dois grupos amostrais: 1) profissionais da Atenção Primária à Saúde (Clínica da Família); 2) profissionais da Atenção Terciária à Saúde (hospital). Foram critérios de inclusão ter, no mínimo, um ano de experiência profissional na instituição e na assistência direta ao paciente como enfermeiro ou técnico de enfermagem. No cenário hospitalar, os dados foram coletados com profissionais que trabalhavam em unidades de internação adulta (clínica médica e cirúrgica). Foram excluídos os participantes afastados do trabalho, sob qualquer tipo de licença. Os participantes foram convidados presencialmente, sem abordagem prévia, no cenário do estudo, sob amostragem por conveniência. Não houve recusa ou desistência de nenhum participante.

### Local do Estudo

Os dados foram coletados em dois cenários (um hospital universitário federal e uma Clínica da Família), ambos localizados na cidade do Rio de Janeiro. O contexto hospitalar apresenta diversas especialidades clínicas, contendo cerca de 550 leitos. A Clínica da Família escolhida foi inaugurada em dezembro de 2010, unidade com 14 equipes da Estratégia Saúde da Família e que atende aproximadamente 52 mil pessoas. A escolha por dois cenários foi sustentada na hipótese de que o contexto pode influenciar a dinâmica de acesso e disseminação de informações, tanto no que diz respeito ao profissional de enfermagem quanto ao perfil dos pacientes de cada esfera de atenção à saúde.

### População

Enfermeiros e técnicos de enfermagem que trabalham no Sistema Único de Saúde, em que o primeiro grupo amostral foi composto por profissionais da Atenção Primária à Saúde atuantes na Clínica da Família, e o segundo grupo amostral foi composto por profissionais da Atenção Terciária à Saúde, atuantes no contexto hospitalar.

### Coleta de Dados

Para a caracterização dos participantes, foi utilizado formulário com questões sociodemográficas. Para o *corpus* analítico, foram empregadas entrevistas semiestruturadas, presenciais e individuais (pesquisadora principal e entrevistado), gravadas em dispositivo eletrônico. O roteiro de perguntas direcionadoras foi elaborado após pesquisa bibliográfica e devido ao aprofundamento temático. As perguntas direcionadoras foram: o que você compreende por *fake news*? Como você percebe esse fenômeno no contexto da saúde e da enfermagem? O que pode ser realizado, no âmbito da enfermagem, para o enfrentamento de *fake news* na saúde? Perguntas circulares foram empregadas à medida que os participantes apresentavam respostas que indicassem a necessidade de aprofundamentos. Os participantes foram orientados a responder às perguntas no tempo que considerassem pertinente, com possibilidade de interromper a entrevista caso considerassem necessário. Como a coleta de dados ocorreu em paralelo ao processo analítico, característica da GT, não foi realizado teste piloto.

Os dados foram coletados entre novembro de 2021 e outubro de 2023, nos cenários da pesquisa, em datas e horários indicados pelos participantes e em ambiente reservado. A coleta foi realizada pela pesquisadora principal (LSC), que é enfermeira, mestre e, à época, doutoranda em enfermagem, sem vínculo com o cenário da pesquisa e sem quaisquer conflitos de interesse com a instituição ou com os profissionais. A pesquisadora principal possui competência com a técnica de coleta e referencial metodológico, a partir de treinamentos junto ao pesquisador orientador (IRS), bem como em pesquisas anteriores.

As entrevistas tiveram duração média de 30 minutos. Por considerar a natureza do método, em que os significados apresentados pelos participantes da pesquisa devem emergir de processos indutivos e dedutivos^(10)^ no decurso da entrevista, sobretudo a partir de perguntas circulares, as entrevistas não foram devolvidas aos participantes para acréscimos ou ajustes.

A coleta de dados foi interrompida a partir da saturação teórica, sob a perspectiva dos autores da GT, mediante processo de coleta e análise simultâneos de dados. Assim, no decurso analítico, os pesquisadores discutiram o processo de identificação dos conceitos/categorias e subcategorias, com densidade teórica capaz de permitir a compreensão da realidade investigada^(9,10)^. Esse processo é favorecido pela utilização de memorandos (anotações reflexivas de campo), recursos analíticos empregados durante e após as entrevistas^(9)^.

Os memorandos permitem direcionar possíveis novas perguntas em entrevistas posteriores, de modo que não houve a necessidade de repetição de entrevistas^(9)^. Ademais, tais recursos permitiram reflexões sobre o desenvolvimento dos conceitos, de modo que auxiliaram na compreensão e consenso sobre a saturação teórica dos dados, devidamente discutida entre os pesquisadores.

### Análise dos Dados

Os dados foram analisados por todos os autores, que eram doutorandos e doutores em enfermagem, além de professores universitários. As entrevistas foram submetidas à análise seguindo as etapas de codificação da GT na perspectiva corbiniana^(9)^, que são aberta, axial e integração. Na codificação aberta, os dados foram segmentados em partes distintas e minuciosamente examinadas, cuja comparação visou captar similaridades entre os dados iniciais (códigos preliminares). A partir da análise comparativa dos códigos preliminares, esses foram agrupados, o que permitiu a delimitação de códigos conceituais, apresentados como representações abstratas de fatos, objetos ou ações percebidos como significativos pelos pesquisadores. O agrupamento dos códigos conceituais por similaridades originou subcategorias e, mediante densidade analítica e conexões entre elas, foi delimitada a categoria^(9,10)^.

As subcategorias foram ordenadas a partir do modelo paradigmático, que considerou três componentes: 1) condições, que tratam dos fatores que influenciam o desenvolvimento do fenômeno investigado; 2) ações-interações, que tratam das estratégias de desenvolvimento e enfrentamento do problema; 3) consequências, que sinalizam as potenciais reações a partir das estratégias implementadas^(9)^.

### Aspectos Éticos

Pesquisa aprovada pelo Comitê de Ética em Pesquisa com Seres Humanos, sob os Pareceres nº 4.970.482, nº 5.036.468 e nº 5.118.815. Os participantes foram esclarecidos quanto à natureza e finalidade da pesquisa, bem como às motivações dos pesquisadores, e assinaram o Termo de Consentimento Livre e Esclarecido. Suas identidades foram mantidas anônimas, pois, ao longo do artigo, foram caracterizadas da seguinte maneira: EH (enfermeiro hospitalar); TEH (técnico de enfermagem hospitalar); EAP (enfermeiro de Atenção Primária); e TEAP (técnico de enfermagem de Atenção Primária). Utilizaram-se os acrônimos seguidos pelo número das suas respectivas entrevistas.

## RESULTADOS

Participaram da pesquisa 25 profissionais de enfermagem, dos quais 17 eram enfermeiros, sendo 11 do cenário hospitalar e seis da Clínica da Família, e oito eram técnicos de enfermagem, dos quais seis eram do cenário hospitalar e dois da Clínica da Família. A média de idade dos profissionais inseridos no contexto hospitalar foi de 35 anos. Já no cenário da Clínica da Família, a média de idade foi de 28 anos.

O tempo médio dos profissionais de enfermagem, no cenário hospitalar, foi de 16 anos, para os técnicos de enfermagem, e de 11 anos, para os enfermeiros. Já os profissionais da Clínica da Família, a média foi de dois anos para os técnicos de enfermagem e quatro anos para os enfermeiros. Todos os enfermeiros possuiam, pelo menos, uma pós-graduação. Apenas três eram técnicos de enfermagem.

Do processo analítico, emergiu o conceito/categoria “Estratégias para o enfrentamento de *fake news* na saúde: uma abordagem complexa sob a perspectiva da enfermagem”, que, sob o emprego do modelo paradigmático, está sustentado nas seguintes subcategorias, assim distribuídas no modelo paradigmático do método: “Operadores de *fake news*: quem são e como evitálos? (condições)”; “Fontes confiáveis: argumentos de autoridade ou autoridade de argumentos?”; “Conhecer para intervir: elementos de identificação de *fake news* (ações-interações)”; “Maneiras de combater *fake news* (consequências)”. Cumpre destacar que os trechos de depoimentos utilizados no decurso do artigo são meramente ilustrativos da contextualização dos resultados, pois os dados brutos (entrevistas) são mais amplos que seus fragmentos.

### Operadores de Fake News: Quem São e Como Evitá-Los?

Esta subcategoria sinaliza, sob a perspectiva dos profissionais de enfermagem, os operadores de *fake news* (aqueles que elaboram e/ou disseminam e/ou consomem notícias falsas) mais expressivos, que se dividem em dois grupos. O primeiro é constituído por pessoas ou fontes de informações próximas, com as quais há interação, como os próprios pacientes, acompanhantes, familiares e vizinhos.

Os profissionais de enfermagem consideraram que as interações entre os emissores de *fake news* e os pacientes, quando estabelecem relações de confiança, implicam interferências na prática profissional e, consequentemente, nos cuidados de enfermagem. Para os enfermeiros e técnicos de enfermagem, as influências de fontes informais, mas confiáveis do ponto de vista dos pacientes, resultam em distorções das práticas de saúde e qualidade do cuidado, cujo cenário é percebido como preocupante para os profissionais de saúde investigados, sobretudo por fragilizar a autoridade de seus argumentos, de modo a implicar a desvalorização de práticas seguras de saúde e de cuidados.

*Eles escutam o vizinho, o site Google, e desmenti-los não é uma tarefa fácil.* (EH11)

*A gente percebe o nosso cotidiano com os pacientes e com nossos familiares. Não acho que exista ninguém atualmente que não conheça um familiar que posta, diariamente, nos grupos, fake news*. (TEAP16)

*E os pacientes são totalmente leigos nos assuntos de saúde. Então, quando eles recebem* (*fake news*) *dos vizinhos ou de um parente, eles acabam acreditando e divulgando essas informações erradas.* (EH4)

*Muitas das vezes, são informações distorcidas do nosso cuidado com nenhuma evidência comprovada, provenientes de um familiar, de um amigo, o que torna essas fake news mais seguras para o paciente. Isso acaba influenciando, porque, como não tem uma relação familiar com ele, tudo que a gente fala é colocado em questionamento.* (EAP13)

*Porque o paciente vem com uma informação que ele acha que tá certa, porque algum conhecido disse. Muitas vezes, nem é profissional da saúde. Falou, ele* (paciente) *acha que tá certo.* (EH3)

*Tem uns que acreditam, mas tem outros que não. Fazem porque a mãe falou, a avó falou* [...] *você vai administrar o medicamento prescrito, aí vem o paciente recusando a administração porque faz mal, porque ele viu no Google que afeta algum sistema ou porque alguém disse.* (EH7)

O segundo grupo percebido pelos profissionais de enfermagem foi formado por personalidades distantes do convívio pessoal dos pacientes e dos próprios profissionais, mas que, de semelhante modo ao grupo anterior, exercem consideráveis influências para o processo de compreensão da realidade, bem como para a construção de significados sobre saúde daqueles que consomem e divulgam *fake news*. Nessa conjuntura, identificaram lideranças políticas, artistas e digitais *influencers* como elementos desse grupo.

Cabe também destacar a percepção dos participantes sobre a politização da informação em saúde, cujo foco pode estar relacionado ao campo de interesses de quem produz e divulga *fake news*, com potenciais comprometimentos da confiança das instituições e profissionais de saúde.

*Na* (pandemia de) *COVID-19, teve posts e mensagens de lideranças falando acerca de assuntos dos quais eles não tinham conhecimento nenhum.* (TEH3)

*Por exemplo, ultimamente, com relação à vacina, algumas pessoas propagam informações que contradizem a sua eficácia, desestimulando a população a se vacinar. Está muito interligado com a política também, eu acho e, com isso, prejudica as políticas de saúde.* (EH6)

*E também tem uma questão política, por exemplo, do governo, disparando informações que não são verdadeiras para benefício próprio.* (EH8)

*A gente recebe um paciente dizendo que viu um vídeo no YouTube* ^®^ *de uma pessoa tal. Muitas vezes, a pessoa que está dando aquela informação lá nem é profissional.* (EAP18)

*Infelizmente, a gente tem uma população que não tem muito estudo, que não entende o processo saúde- doença e tudo que vê na internet acredita e acaba disseminando. Elas deixam se levar por influencers que aparecem na mídia. Por exemplo, artistas, políticos que eles possuem admiração. Quando pessoas nesta posição acabam falando algo que desconhecem, eles possuem o papel de influenciar pessoas leigas.* (TEH3)

Os profissionais de enfermagem reconhecem que os operadores de *fake news* estabelecem tais interações de forma expressiva no ambiente virtual a partir das mídias sociais. Os significados atribuídos pelos participantes a essas plataformas são relacionados aos perigos que oferecem, especialmente em decorrência da dinâmica que assumem nos processos de divulgação e acesso de *fake news.*

*A internet, o Instagram* ^®^ *, que é surreal, é muito perigoso, e o WhatsApp* ^®^ *, que é mais surreal ainda.* (EH24)

*Principalmente as redes sociais. O Instagram* ^®^ *, o Twitter* ^®^ (“X”)*, acho que propagam muitas fake news.* (EH23)

*Nos* últimos *tempos, as fake news ganharam força por conta do WhatsApp* ^®^ *, né?!* (TEAP20);

*Notícias falsas estão sendo repassadas* [...] *através das redes sociais.* (TEH12)

### Fontes Confiáveis: Argumentos de Autoridade ou Autoridade de Argumentos?

Os profissionais de enfermagem atribuem significados que sinalizam confiabilidade para fontes oficiais de instituições consolidadas, como Ministério da Saúde, Secretarias Municipais de Saúde e nas Normas Técnicas oriundas desses contextos. Reconhecem a confiabilidade de fontes jornalísticas, sobretudo aquelas que corroboram as fontes governamentais.

O reconhecimento das fontes confiáveis significadas pelos profissionais de enfermagem está centrado no argumento da autoridade que tais fontes representam. Nesse sentido, valorizam órgãos especializados, como Fundação Oswaldo Cruz e Agência Nacional de Vigilância Sanitária. Ademais, os participantes sinalizaram a importância da facilidade de acesso à informação a partir de mídias sociais utilizadas pelos órgãos governamentais e especializados.

*Eu acredito que deve buscar as informações das Normas Técnicas, que são divulgadas pela Secretaria de Saúde, ficar atenta* às *redes da prefeitura, participar das capacitações que são oferecidas.* (EAP17)

*Eu acho que, hoje em dia, está tudo muito fácil. Até o próprio Ministério da Saúde, a Anvisa tem Instagram* ^®^ *para desmentir as informações nas redes. Então, esses* órgãos *entraram nas redes para educar. Eu tento entrar através desses* órgãos*.* (EH6)

*O Ministério da Saúde, para mim, é uma fonte segura.* Às *vezes, quando é uma notícia de saúde que passou nos jornais, que eles dizem, né?! Procurar a fonte jornalística mais segura.* (EH11)

*Eu acho que são os* órgãos *do governo, o Ministério da Saúde, a Fiocruz. Para mim, são eles que têm um grupo de especialistas que vão estudar e conseguir passar as medidas corretas.* (TEAP20)

Em perspectiva descentralizada, os profissionais de enfermagem atribuíram confiabilidade às fontes que se constituíram nas interações do cotidiano laboral, tais como chefias/lideranças de enfermagem e demais enfermeiros. Entretanto, revelaram, também, certo grau de ceticismo em relação à busca por informações junto a profissionais que, embora por eles reconhecidos como referências de conhecimento, nem sempre foram percebidos, de forma inequívoca, como fontes seguras de informação.

*A gente busca de alguma forma. Quando tem alguma coisa diretamente relacionada ao hospital, a gente pergunta à chefia. Pelo menos, eu pergunto.* (TEH5)

*Eu considero os conhecimentos do enfermeiro como fontes importantes.* (TEAP16)

*Então, eu acho que elas devem desconfiar, a princípio, e depois, sempre escutar as pessoas que são referências naquela área, quem estuda o assunto. Os enfermeiros podem ser fontes confiáveis.* (EH2)

Também foram elencadas pelos participantes como fontes seguras de informação aquelas consideradas veículos tradicionais de comunicação, como rádio, televisão e jornais, cuja confiabilidade está na autoridade do argumento que os enfermeiros e técnicos de enfermagem compreendem que é estabelecida a partir do compromisso e da capacidade de investigação desses veículos.

*Buscando em canais de notícias fidedignas, como os jornais que já possuem um processo de comprovar a informação*. (EH25)

*Meios mais tradicionais de informação como jornais, mesmo que online, mas que tragam o nome e a referência de onde saiu aquela informação.* (EH14)

*Na internet, você posta o que você quer. O jornalismo, na televisão, precisa, de fato, de embasamento para passar aquela informação para você. Além de noticiar, ele vai no local, ele faz as perguntas. Existe um processo de investigação.* (EH26)

*Sempre busco informações nas bases de dados e também de telejornais, porque eles já possuem mecanismos de checagem da informação. Depois da pandemia de COVID-19, acho que se intensificou ainda mais.* (EAP19)

Para os enfermeiros entrevistados, são fontes confiáveis de informações aquelas relacionadas diretamente à ciência, a partir de bases de dados especializadas, como LILACS, BDENF, PubMed, além de ferramentas mais genéricas, como o *Google* ^®^. Nesse contexto, significam as revistas científicas como fontes confiáveis para abordagens específicas, como discussão de casos.

*A base de dados da saúde mesmo. O LILACS, BDENF, PubMed são as que dão mais respaldo pra gente mesmo, e não o Google* ^®^ *.* (EAP14)

*Eu acredito que o enfermeiro tem que se basear em informações de fontes seguras, como as bases de dados, as bibliotecas virtuais e revistas científicas.* (EH04)

*Buscar artigos nas bases de dados, como o LILACS e BDENF, que concentram ali acervo de revistas científicas.* (EH06)

*Também temos acesso aos artigos, então, sempre que há discussão de casos, buscamos as revistas científicas, as bases de dados.* (EAP17)

### Conhecer Para Intervir: Elementos de Identificação de Fake News

Para os participantes do estudo, *fake news* apresentam indícios em sua estrutura que permitem identificar a sua natureza mentirosa. Dessa maneira, algumas ações foram apontadas, como a necessidade de se verificar a fonte da informação e confrontá-la com outras fontes consideradas seguras.

Conforme o exposto, para os profissionais de enfermagem, as *fake news* podem apresentar, no conjunto de seus dados informativos, uma parte verdadeira que é associada com uma parte falsa. Nesse sentido, mencionam a autoria, o canal de comunicação em que a notícia é veiculada e o método utilizado como indicadores que garantem a confiabilidade da informação acessada e disseminada.

*Essas notícias são como telefones sem fio. Possuem uma mínima parte de verdade, mas quem vai repassando vai sempre aumentando com mentiras.* (EH8)

*Às vezes, é citada uma coisa, e isso, na* área *da saúde, acontece muito. As pessoas veem que algo está sendo estudado, e já misturam os dados de outros estudos, e distorcem a informação.* (EH22)

*Então, observar o autor daquela matéria, procurar em mais de uma fonte. Se for vídeo, observar a qualidade do vídeo, se ele foi editado.* (EH23)

*Acreditando que pesquisar, observar quem foi a pessoa que escreveu aquela informação. Verificar se ela é uma profissional que realmente conhece sobre aquele assunto*. (TEH21)

*Além disso, ter muita atenção ao ler essas informações falsas, normalmente, sempre vem com um título sensacionalista, com um nome conhecido que deixa a gente sempre na dúvida.* (EAP15)

*Eu acho que elas devem, a princípio, desconfiar. Depois, sempre escutar as pessoas que são referências naquela* área*, quem estuda o assunto.* (EH2)

Entretanto, os entrevistados, apesar de sinalizarem esses elementos de identificação, consideraram que as *fake news* são informações de difícil reconhecimento, como demonstram os trechos a seguir:

*Quando vemos as notícias nas redes, fica difícil você distinguir o que é mentira ou verdade naquela informação, porque, cada dia que passa, elas estão mais bem elaboradas.* (EH1)

*Às vezes, a gente fica com dúvida quando se depara com essas informações falsas. Porque, hoje em dia, os recursos também estão bem avançados* (TEAP20)

*Eu não vou dizer que é uma tarefa fácil. Nem sempre, à primeira vista, a gente consegue identificar.* (EAP19)

### Maneiras de Combater Fake News

Esta subcategoria contempla os dados apresentados pelos participantes do estudo nas ações que, segundo eles, resultam no combate à disseminação de *fake news* na saúde, mesmo no contexto de trabalho da enfermagem, em especial junto aos pacientes. Para tal finalidade, os dados revelaram a importância da educação em saúde como principal estratégia utilizada pelos profissionais.

Os recursos apontados pelos depoentes apresentam diferentes possibilidades de enfrentamento de *fake news*, sobretudo aquelas centradas na orientação e em recursos visuais, a partir da elaboração de cartilhas com foco nos problemas de saúde.

*Eu acho que a* única *maneira é orientar pacientes e acompanhantes. É o que a gente tem feito aqui trazendo uma pessoa que trabalhe naquela* área *para explicar.* (TEH07)

*Acho que as cartilhas informativas podem ser uma opção. Acredito que essa educação sobre informações falsas é interesse de todo mundo.* (EH2)

*Orientação diariamente dos pacientes, dos acompanhantes. E acho importante que essas ações de educação envolvam toda comunidade, os agentes de saúde, o representante comunitário, as escolas*. (TEAP16)

*Eu normalmente tenho conversado, orientado quando encontro pacientes ou familiares que acreditam em notícias que são propagadas falsamente nas redes sociais. Nem sempre eles escutam, mas o meu papel de orientar, eu faço.* (EH1)

*Eu acho que educação em saúde, que é uma coisa que é muito presente, inclusive na Atenção Primária, também. Acho que a gente falar e informar as pessoas, levar conhecimento* às *pessoas. Acredito que seja uma ferramenta fundamental basicamente para se combater fake news.* (EAP15)

Outro recurso pedagógico sinalizado está na necessidade de campanhas de conscientização com o objetivo de combate aos mitos e tabus que contribuem para a propagação e reiteração de *fake news*.

*Eu acho que deveria ter campanhas de conscientização em relação ao compartilhamento dessas informações, porque essas informações acabam tendo essa magnitude toda porque há pessoas que compartilham.* (EH4)

*Acho que campanhas dentro do hospital para poder conscientizar os pacientes em relação a certos cuidados. Eu penso que, sempre que possível, deve haver ações de educação: podem ser palestras, rodas de conversas* [...] *acho que a gente deve se aproximar desse paciente, tentando entender as dúvidas e elucidar ponto a ponto, como eu faço quando me deparo com situações de falta de informação.* (TEH3)

*Uma ação interessante que eu acho que pode ser feita, igual é feita na vacinação, são as campanhas de conscientização, porque pode sensibilizar e alertar as pessoas que fake news geram problemas.* (EAP14)

Os enfermeiros da área hospitalar mencionaram, como estratégia de enfrentamento de *fake news*, o desenvolvimento e a valorização de perfil de checagem de informações nas redes sociais, com destaque para o *Workplace* ^®^.

*Eu penso que deve ter uma fonte segura, uma plataforma, sei lá, onde seja possível você replicar a informação e, no final, ela dizer se é verdadeira ou não.* (EH6)

*Vejo que poderia ser criado no público algo como Workplace* ^®^ *. É similar ao Facebook* ^®^ *, porém com intuito de disseminar ações, informações seguras na instituição. Acho uma ideia interessante que poderia ser utilizada como estratégia, pois seria como uma rede social do hospital e que daria para gerenciar as informações pelos profissionais.* (EH08)

Ademais, os participantes ressaltaram a educação permanente como estratégia importante junto aos profissionais de enfermagem, a partir da realização de cursos ou palestras, com intuito de capacitá-los mediante conhecimentos e atitudes, a fim de transladar tais conhecimentos aos pacientes.

*Acho que nós profissionais precisamos de cursos de capacitação, cursos ou palestras que elucidem dúvidas dos assuntos que usualmente são apontados nas fake news.* (EH4)

*Essas capacitações são importantes porque nos dá segurança. Nos faz ter uma reflexão do nosso cotidiano de trabalho; traz novas trocas. Isso faz com que a gente se sinta mais preparado para enfrentar essas notícias quando o usuário nos traz.* (EAP17)

Portanto, do conjunto dos princípios/subcategorias, emergiu a teoria substantiva, pautada na complexidade, que sinaliza a multidimensionalidade para o enfrentamento das *fake news* na saúde, a partir da perspectiva da enfermagem. O objetivo é reconhecer as conexões entre os operadores das *fake news*, as fontes de informação e suas implicações simbólicas, entre argumentos de autoridade e autoridades do argumento, bem como as maneiras múltiplas de combater as notícias falsas na saúde.

## DISCUSSÃO

O contexto de onde emergem *fake news* influencia tanto ou mais do que a própria informação falsa divulgada. Nesse sentido, lideranças políticas e sanitárias devem assumir o compromisso com políticas públicas e estratégias mobilizadoras para um conhecimento capaz de romper a lógica pautada na pós-verdade, mediante inclinações limitadas ao campo das emoções^(8,11)^. Tal realidade é fortalecida por bolhas virtuais, impulsionadas por tecnologias de comunicação, como *WhatsApp* ^®^, *Facebook* ^®^ *, Instagram* ^®^ *, “X”* ^®^ e tantas outras. Em conjunto, tais mídias estimulam interações restritas às perspectivas comuns aos grupos de pertencimento virtuais, de modo a reiterar o processo não dialético de conhecimento em grupos restritos^(4,11,12)^.

Na perspectiva da complexidade^(1)^, a relação de interdependência entre grupo e integrantes não se estabelece apenas pelo princípio hologramático, no qual o todo contém as partes e as partes representam o todo. Para o pensamento complexo, a dinâmica de interação entre essas dimensões faz emergir, sobretudo, o princípio do circuito-recursivo, cujos atores são produtores e produtos de si mesmos. Assim, a reiteração do pensamento comum que reifica as *fake news* está pautada na qualidade das interações entre as partes e o todo, cuja relação de confiança exerce reciprocidade entre ambos, conformando-se em fator preponderante às ações motivadoras para o consumo e compartilhamento de *fake news* ^(13)^.

A interação entre conceitos e princípios desta pesquisa corrobora o princípio do circuito-recursivo, por estabelecer sobre os emissores de *fake news* um efeito de produto-produtor, pois, ao mesmo tempo em que emitem notícias falsas, são também consumidores-dependentes delas^(1)^. Nesse processo cíclico, a literatura científica reitera que, no contexto da saúde, tratando-se de *fake news*, o discurso de autoridade prevalece sobre a autoridade do discurso^(14)^, razão pela qual instituições de saúde e de pesquisa, assim como os próprios enfermeiros, devem estabelecer estratégias que permitam alcançar projeções de valorização da ciência^(15)^. Para tanto, tais atores devem ser compreendidos como autoridades epistêmicas, facilitadoras do acesso à informação para a construção do conhecimento^(16,17)^, que visa, entre outros objetivos, o enfrentamento das *fake news* na ciência e na saúde^(18)^.

Esta pesquisa corrobora o que tem sido abordado na literatura científica sobre a magnitude das influências dos atores (lideranças e instituições), que conformam a perspectiva da autoridade do discurso sobre a disseminação e/ou combate de *fake news*. Todavia, o nível de conhecimento científico das pessoas que consomem e compartilham *fake news* também é um fator interveniente nessa realidade^(19)^, de modo a reiterar a necessidade de melhores investimentos na formação científica do enfermeiro^(20)^. Nessa conjuntura, tem-se que o enfermeiro, ao longo de sua formação, deve ser preparado para desenvolver competências de liderança, entre as quais está a condição precípua de mobilizar esforços para um propósito comum, sob uma lógica capaz de motivar, pelo exemplo, o que inclui a valorização da ciência. Assim, tem-se que é papel do enfermeiro se manter informado e atualizado sobre as matérias que poderão influenciar os seus processos decisórios, o que envolve a necessidade de vigilância epistêmica e proficiência científica para saber interpretar dados e decodificar informações que se supõem científicas^(21)^.

Diante do exposto, é possível inferir que o enfrentamento da desinformação envolve a própria complexidade na gestão do conhecimento, como a capacidade para consumir, decodificar, comunicar e avaliar a informações científicas que visam gerar conhecimentos, cujas consequências poderão refletir comportamentos que afetam a saúde. Portanto, faz-se necessário, também, saber a dinâmica e a procedência das informações consumidas^(17)^, bem como a assunção da necessidade de proficiência tecnológica e científica para o acesso, consumo e disseminação de informações verdadeiras.

Algumas ações podem compor a vigilância no combate à desinformação ocasionada por *fake news* no âmbito da saúde. Os dados desta pesquisa corroboram, também, indicativos de vigilância epistêmica da informação, pois, no jornalismo, por exemplo, a garantia da integridade da informação cabe ao próprio contexto jornalístico, seja pelo profissional, pelo veículo de divulgação ou por associações relacionadas ao compromisso com a veracidade das notícias na imprensa. Entretanto, outras medidas podem fazer a diferença, como a verificação da data da informação divulgada^(22)^, pois o espaço temporal pode fazer com que a informação obsoleta configure uma *fake news* quando divulgada como conjunto de dados atuais. Ademais, a velocidade com que as *fake news* se espalham é maior que a dinâmica de estratégias para combatê-las, dentre as quais estão as agências de checagem jornalísticas, que funcionam de acordo com a demanda dos usuários, que sinalizam a necessidade de verificar essas notícias^(23)^.

A utilização de recursos tecnológicos em ações de educação em saúde configura, também, importante estratégia para o combate à desinformação. Todavia, cabe ressaltar a importância de os profissionais de enfermagem buscarem esses recursos e disponibilizar informações coerentes, de qualidade, fundamentadas cientificamente^(24)^. Para tanto, o processo de comunicação da ciência, especialmente no âmbito da saúde, deve ser uma competência a ser, constantemente, desenvolvida pelos profissionais de enfermagem^(20)^.

Tratando-se das fontes, os resultados desta pesquisa são corroborados por evidências que sinalizam a natureza das fontes de informações como elemento que integra a multidimensionalidade dos acessos e disseminação de *fake news* na saúde, a partir das mídias sociais. Em estudo realizado pela *Iowa State University* com estudantes universitários, demonstrou-se que a maioria (69%) dos participantes conseguiu determinar com sucesso a veracidade das publicações sobre saúde em mídias sociais. Porém, destacou que, entre os elementos que direcionaram a capacidade de julgamento dos estudantes, estão principalmente a credibilidade conferida à fonte, à aparência e ao estilo do design das postagens^(19)^.

Em uma perspectiva complexa, a disseminação de *fake news* na saúde deve ser compreendida sob análise de conjuntura que seja capaz de destacar as nuances contextuais que envolvem elementos socioeconômicos e culturais^(25)^. Tal entendimento é corroborado por pesquisa realizada com 5.307 entrevistados, em seis países da África Subsaariana, cujos dados destacaram que, embora parte expressiva das *fake news* sobre saúde seja disseminada acidentalmente, há uma parte considerável de indivíduos que compartilham propositalmente tais informações. Porém, o mais significativo desses dados está nos determinantes individuais, os quais sinalizaram que pessoas mais velhas, do sexo masculino, mais propensas a assumirem riscos, são os atores mais inclinados à prática de compartilhamento de informações falsas sobre saúde^(25)^.

Os dados desta pesquisa são, portanto, corroborados pela literatura, notadamente em relação à multidimensionalidade que envolve o consumo e a disseminação de *fake news* na área da saúde, a partir da importância do contexto, das fontes e da reiteração das notícias^(8,16,19,24)^. Todavia, eles se destacam por apresentar uma perspectiva complexa da enfermagem sobre a temática, especialmente por ser esta a profissão que ocupa o maior número de postos de trabalho no setor de saúde em todos os sistemas do planeta, bem como por ser estratégica para o combate à desinformação na área^(8)^.

As limitações da pesquisa estão relacionadas ao campo epistêmico, posto que a natureza dos participantes inseridos somente no contexto público de saúde pode, por exemplo, conformar-se em significados específicos sobre *fake news* nas áreas da saúde e da enfermagem.

## CONCLUSÃO

Os significados desvelados pelos profissionais de enfermagem consideraram a complexidade do combate às *fake news* na saúde e na própria enfermagem, evidenciando que a disseminação dessas informações ocorre por múltiplas vias e está inserida em dinâmicas sociais, políticas e tecnológicas. Para os enfermeiros e técnicos de enfermagem, independentemente do cenário de atuação, há desafios significativos ao lidar com pacientes influenciados por fontes não confiáveis, sejam elas familiares, redes sociais ou figuras públicas. Ademais, os resultados desta pesquisa alcançaram o objetivo do estudo, ao sinalizarem a importância do desenvolvimento de mecanismos institucionais de checagem e da promoção de campanhas educativas como estratégias essenciais para minimizar os impactos das *fake news* no cuidado à saúde.

## DISPONIBILIDADE DE DADOS

Todo o conjunto de dados que dá suporte aos resultados deste estudo foi publicado no próprio artigo.

# Translation: Estrategias para hacer frente a las fake news en el ámbito de la salud: un enfoque complejo desde la perspectiva de la enfermería*

**DOI:** 10.1590/1980-220X-REEUSP-2025-0111es

**Authors:** Luana dos Santos Costa, Carla Arena Aparecida Ventura, Sonia Silva Marcon, Sabrina da Costa Machado Duarte, Thiago Privado da Silva, Ana Larissa Araujo Nogueira, Ítalo Rodolfo Silva

**Affiliations:** 1 Universidade Federal do Rio de Janeiro, Escola de Enfermagem Anna Nery, Rio de Janeiro, RJ, Brasil.; 2 Universidade de São Paulo, Escola de Enfermagem de Ribeirão Preto, Ribeirão Preto, SP, Brasil.; 3 Universidade Estadual de Maringá, Maringá, PR, Brasil.

**Keywords:** Enfermería, Salud, Desinformación, Enfermeras Practicantes, Gestión del Conocimiento

## Abstract

**Objetivo:**

Revelar las estrategias para hacer frente a las fake news en el contexto de la salud y la enfermería desde la perspectiva de los profesionales de enfermería.

**Método:**

Investigación cualitativa, cuyos marcos teórico y metodológico fueron, respectivamente, la Teoría de la Complejidad y la Grounded Theory. Participaron 25 profesionales de enfermería de un hospital público y de una Clínica de Familia de la ciudad de Río de Janeiro. Se realizaron entrevistas semiestructuradas entre 2021 y 2023.

**Resultados:**

Los significados revelados por los profesionales de enfermería pusieron de manifiesto la complejidad de la lucha contra las fake news (noticias falsas), evidenciando que la difusión de esta información ocurre por múltiples vías y está insertada en dinámicas sociales, políticas y tecnológicas. Los resultados señalaron la importancia del desarrollo de mecanismos institucionales de verificación y la promoción de campañas educativas como estrategias para minimizar los impactos de las noticias falsas en la atención de la salud.

**Conclusión:**

La perspectiva compleja del estudio señala que la lucha contra las noticias falsas en la salud y la enfermería exige enfoques multidimensionales que combinen educación, regulación y valoración de la ciencia en la construcción de conocimiento confiable.

## INTRODUCCIÓN

Desde la perspectiva posmoderna, la comprensión ontológica del ser privilegia una realidad multifacética, concebida como dinámica y en constante transformación. En este sentido, el lenguaje asume un papel central en la construcción de la realidad, de modo que cualquier pretensión de alcanzar la verdad objetiva se configura como un metadiscurso^(1,2)^. Así, surgen nuevas formas narrativas y metodológicas, acordes con los desafíos de los nuevos tiempos, en las que las noticias falsas, conocidas como *fake news,* a pesar de ser mecanismos habitualmente utilizados en los enfrentamientos políticos, pasan a incidir también sobre las narrativas que impregnan el contexto de la salud pública^(3)^.

En los últimos años, se ha observado la expansión de las noticias falsas en los movimientos antivacunas^(4)^, lo que ha tenido como consecuencia el resurgimiento de enfermedades consideradas, hasta entonces, erradicadas. Se trata de publicaciones en redes sociales (*posts*), audios y videos que se vuelven virales en Internet y validan la percepción engañosa de que la vacuna es prescindible. Un hito importante en este proceso, según la Organización Mundial de la Salud, fue la pandemia de COVID-19, que puso de manifiesto las noticias falsas como un fenómeno global de importancia para la salud pública^(5)^.

Actualmente, la dinámica de acceso y difusión de la información destinada a la construcción y el uso del conocimiento está influenciada por las Tecnologías de la Información y la Comunicación (TIC). Las TIC establecen, asimismo, formas nuevas y definidoras de patrones y conductas de conocimiento que afectan el proceso de trabajo y los servicios de salud, ya que influyen en las acciones de las personas que acceden a dichos servicios^(6)^.

El proceso de trabajo de la enfermería se relaciona con la transitoriedad, la discontinuidad y el caos de los tiempos posmodernos. Así, se han configurado nuevos principios organizacionales que implican, además, la necesidad de que el equipo de enfermería desempeñe múltiples funciones, de manera descentralizada, con flexibilidad, liderazgo, asunción de riesgos, comunicación efectiva y habilidades relacionales, entre otras^(7)^. En este contexto, las TIC influyen en el proceso de gestión del conocimiento de la enfermería, desde el acceso y la difusión de información entre el equipo hasta la toma de decisiones en la atención directa al paciente^(6)^. En la dinámica de este fenómeno complejo se encuentra la realidad de las *fake news* como un problema global que afecta a todos los sectores del tejido social, y la enfermería no es una excepción.

Ante lo expuesto, se entiende que el proceso de trabajo de la enfermería puede verse afectado por las noticias falsas, debido a su influencia en los elementos que envuelven la realidad laboral de estos profesionales, como objeto, agente, instrumento, finalidad, método y producto, lo que conlleva modificaciones y posibles perjuicios en todas las dimensiones de su trabajo. En este contexto, las noticias falsas deben considerarse un desafío para la elaboración de estrategias efectivas de orientación en salud para la población y para la gestión del conocimiento en el contexto laboral, así como para la competencia política del enfermero^(8)^.

De este modo, se sostiene el supuesto de que los significados que los enfermeros atribuyen a las noticias falsas pueden orientar sus conductas al enfrentar este importante problema de salud pública y, de hecho, influir en el proceso de trabajo de la enfermería a partir de la gestión del conocimiento involucrado en la dinámica de acceso, difusión y consumo de información verificada, para la utilización de bases científicas pertinentes en el contexto en el que ejercen la atención, sobre todo para la orientación de pacientes que pueden consumir y difundir noticias falsas.

Desde una perspectiva sistémica, esta investigación contempla el paradigma de la complejidad al reconocer que el conocimiento es un proceso relacional, en el cual la Teoría de la Complejidad considera que el proceso de conocer resulta de la articulación entre las dimensiones neurobiológicas del cerebro y los elementos socioculturales que conforman la sociedad^(1)^. Por lo tanto, no se puede comprender el fenómeno de las noticias falsas en la salud y la enfermería sin prestar atención a las perspectivas presentadas por los profesionales de enfermería.

Ante lo expuesto, el estudio tiene como objetivo revelar las estrategias para hacer frente a las *noticias falsas* en el contexto de la salud y la enfermería desde la perspectiva de los profesionales de enfermería.

## MÉTODO

### Tipo de Estudio

Investigación cualitativa, cuyos marcos teórico y metodológico fueron, respectivamente, la Teoría Fundamentada (*Grounded Theory* - GT)^(9,10)^ y la Teoría de la Complejidad^(1)^. Para la confiabilidad analítica de la investigación, se emplearon las directrices del *Equator*, en particular la guía *COnsolidated criteria for REporting Qualitative research*.

### Criterios de Selección

Participaron en el estudio enfermeros y técnicos de enfermería, distribuidos en dos grupos de muestra: 1) profesionales de la Atención Primaria de Salud (Clínica de Familia); 2) profesionales de la Atención Terciaria de Salud (hospital). Los criterios de inclusión fueron tener, como mínimo, un año de experiencia profesional en la institución y en la atención directa al paciente como enfermero o técnico de enfermería. En el entorno hospitalario, los datos se recopilaron con profesionales que trabajaban en unidades de hospitalización de adultos (clínica médica y quirúrgica). Se excluyó a los participantes ausentes del trabajo, bajo cualquier tipo de licencia. Se invitó a los participantes en persona, sin contacto previo, en el entorno del estudio, mediante muestreo por conveniencia. No hubo rechazo ni desistimiento de ningún participante.

### Lugar del Estudio

Los datos se recopilaron en dos entornos (un hospital universitario federal y una Clínica de Familia), ambos ubicados en la ciudad de Río de Janeiro. El entorno hospitalario cuenta con diversas especialidades clínicas y dispone de cerca de 550 camas. La Clínica de Familia elegida fue inaugurada en diciembre de 2010, una unidad con 14 equipos de la Estrategia de Salud de la Familia que atiende a aproximadamente 52 000 personas. La elección de dos entornos se basó en la hipótesis de que el contexto puede influir en la dinámica de acceso y difusión de la información, tanto en lo que respecta al personal de enfermería como al perfil de los pacientes de cada ámbito de atención de salud.

### Población

Enfermeros y técnicos de enfermería que trabajan en el Sistema Único de Salud, en el que el primer grupo de la muestra estuvo compuesto por profesionales de la Atención Primaria de Salud que trabajan en la Clínica de Familia, y el segundo grupo de la muestra estuvo compuesto por profesionales de la Atención Terciaria de Salud, que trabajan en el contexto hospitalario.

### Recolección de Datos

Para la caracterización de los participantes, se utilizó un formulario con preguntas sociodemográficas. Para el *corpus*analítico, se emplearon entrevistas semiestructuradas, presenciales e individuales (investigadora principal y entrevistado), grabadas en un dispositivo electrónico. El guion de preguntas orientativas se elaboró tras una investigación bibliográfica y debido a la profundización temática. Las preguntas orientativas fueron: ¿qué entiende usted por *fake news*? ¿Cómo percibe este fenómeno en el contexto de la salud y la enfermería? ¿Qué se puede hacer, en el ámbito de la enfermería, para hacer frente a las *noticias falsas* en la salud? Se emplearon preguntas circulares a medida que los participantes presentaban respuestas que indicaban la necesidad de profundizar. Se indicó a los participantes que respondieran a las preguntas en el tiempo que consideraran pertinente, con la posibilidad de interrumpir la entrevista si lo consideraban necesario. Dado que la recopilación de datos se llevó a cabo en paralelo al proceso analítico, característica del GT, no se realizó una prueba piloto.

Los datos se recopilaron entre noviembre de 2021 y octubre de 2023, en los escenarios de la investigación, en las fechas y horarios indicados por los participantes y en un entorno privado. La recopilación fue realizada por la investigadora principal (LSC), que es enfermera, tiene una maestría y, en ese momento, era doctoranda en enfermería, sin vínculos con el escenario de la investigación y sin ningún conflicto de intereses con la institución o con los profesionales. La investigadora principal posee competencia en la técnica de recopilación y el marco metodológico, a partir de capacitaciones con el investigador orientador (IRS), así como en investigaciones anteriores.

Las entrevistas tuvieron una duración media de 30 minutos. Dada la naturaleza del método, en el que los significados presentados por los participantes de la investigación deben surgir de procesos inductivos y deductivos^(10)^ durante el transcurso de la entrevista, sobre todo a partir de preguntas circulares, las entrevistas no se devolvieron a los participantes para que realizaran añadidos o ajustes. La recopilación de datos se interrumpió a partir de la saturación teórica, desde la perspectiva de los autores de la GT, mediante un proceso de recopilación y análisis simultáneos de datos. Así, durante el proceso analítico, los investigadores discutieron el proceso de identificación de los conceptos/categorías y subcategorías, con una densidad teórica capaz de permitir la comprensión de la realidad investigada^(9,10)^. Este proceso se ve favorecido por el uso de memorandos (anotaciones reflexivas de campo), recursos analíticos empleados durante y después de las entrevistas^(9)^.

Los memorandos permiten orientar posibles nuevas preguntas en entrevistas posteriores, de modo que no hubo necesidad de repetir entrevistas^(9)^. Además, dichos recursos permitieron reflexiones sobre el desarrollo de los conceptos, de modo que ayudaron a la comprensión y al consenso sobre la saturación teórica de los datos, debidamente discutida entre los investigadores.

### Análisis de los Datos

Los datos fueron analizados por todos los autores, quienes eran doctorandos y doctores en enfermería, además de profesores universitarios. Las entrevistas se sometieron a análisis siguiendo las etapas de codificación de la GT desde la perspectiva corbiniana^(9)^, que son: abierta, axial e integración.

En la codificación abierta, los datos se segmentaron en partes distintas y se examinaron minuciosamente, y su comparación tuvo como objetivo captar similitudes entre los datos iniciales (códigos preliminares). A partir del análisis comparativo de los códigos preliminares, estos se agruparon, lo que permitió delimitar códigos conceptuales, presentados como representaciones abstractas de hechos, objetos o acciones percibidos como significativos por los investigadores. La agrupación de los códigos conceptuales por similitudes dio lugar a subcategorías y, mediante la densidad analítica y las conexiones entre ellas, se delimitó la categoría^(9,10)^.

Las subcategorías se ordenaron a partir del modelo paradigmático, que consideró tres componentes: 1) condiciones, que tratan de los factores que influyen en el desarrollo del fenómeno investigado; 2) acciones-interacciones, que tratan de las estrategias de desarrollo y afrontamiento del problema; 3) consecuencias, que señalan las reacciones potenciales a partir de las estrategias implementadas^(9)^.

### Aspectos Éticos

Investigación aprobada por el Comité de Ética en Investigación con Seres Humanos, bajo los dictámenes n.º 4.970.482, n.º 5.036.468 y n.º 5.118.815. Se informó a los participantes sobre la naturaleza y finalidad de la investigación, así como sobre las motivaciones de los investigadores, y firmaron el Formulario de Consentimiento Libre e Informado. Se mantuvo el anonimato de sus identidades, ya que, a lo largo del artículo, se les identificó de la siguiente manera: EH (enfermero hospitalario); TEH (técnico de enfermería hospitalaria); EAP (enfermero de atención primaria); y TEAP (técnico de enfermería de atención primaria). Se utilizaron los acrónimos seguidos del número de sus respectivas entrevistas.

## RESULTADOS

Participaron en la investigación 25 profesionales de enfermería, de los cuales 17 eran enfermeros, siendo 11 del ámbito hospitalario y seis de la Clínica de Familia, y ocho eran técnicos de enfermería, de los cuales seis eran del ámbito hospitalario y dos de la Clínica de Familia. La edad promedio de los profesionales del ámbito hospitalario fue de 35 años. En el ámbito de la Clínica de Familia, la edad promedio fue de 28 años.

La antigüedad promedio de los profesionales de enfermería en el ámbito hospitalario fue de 16 años para los técnicos de enfermería y de 11 años para los enfermeros. En cuanto a los profesionales de la Clínica de Familia, el promedio fue de dos años para los técnicos de enfermería y de cuatro años para los enfermeros. Todos los enfermeros contaban con, al menos, un posgrado. Solo tres eran técnicos de enfermería.

Del proceso analítico surgió el concepto/categoría “Estrategias para hacer frente a las *fake news* en la salud: un enfoque complejo desde la perspectiva de la enfermería”, que, bajo el empleo del modelo paradigmático, se sustenta en las siguientes subcategorías, distribuidas así en el modelo paradigmático del método: “Operadores de *fake news*: ¿quiénes son y cómo evitarlos? (condiciones)”; “Fuentes confiables: ¿argumentos de autoridad o autoridad de los argumentos?”; “Conocer para intervenir: elementos de identificación de noticias falsas (acciones-interacciones)”; “Formas de combatir las noticias falsas (consecuencias)”. Cabe destacar que los fragmentos de testimonios utilizados a lo largo del artículo son meramente ilustrativos de la contextualización de los resultados, ya que los datos brutos (entrevistas) son más amplios que sus fragmentos.

### Operadores de Noticias Falsas: ¿Quiénes Son y Cómo Evitarlos?

Esta subcategoría señala, desde la perspectiva de los profesionales de enfermería, a los operadores de noticias falsas (aquellos que elaboran y/o difunden y/o consumen noticias falsas) más significativos, que se dividen en dos grupos. El primero está formado por personas o fuentes de información cercanas, con las que hay interacción, como los propios pacientes, acompañantes, familiares y vecinos.

Los profesionales de enfermería consideraron que las interacciones entre los emisores de noticias falsas y los pacientes, cuando establecen relaciones de confianza, implican interferencias en la práctica profesional y, en consecuencia, en los cuidados de enfermería. Para los enfermeros y técnicos de enfermería, las influencias de fuentes informales, pero confiables desde el punto de vista de los pacientes, dan lugar a distorsiones en las prácticas de salud y la calidad de la atención, cuyo escenario se percibe como preocupante para los profesionales de la salud investigados, sobre todo porque debilita la autoridad de sus argumentos, lo que implica una desvalorización de las prácticas seguras de salud y de atención.

*Escuchan al vecino, al sitio de Google, y desmentirlos no es una tarea fácil.* (EH11)

*Nos damos cuenta de nuestro* día a día con *los pacientes y con nuestros familiares. No creo que haya nadie hoy en* día *que no conozca a un familiar que publique, a diario, en los grupos, noticias falsas*. (TEAP16)

*Y los pacientes son totalmente legos en temas de salud. Entonces, cuando reciben* (*noticias falsas*) *de los vecinos o de un pariente, terminan creyendo y difundiendo esa información errónea.* (EH4)

*Muchas veces, se trata de información distorsionada sobre nuestra atención, sin ninguna evidencia comprobada, proveniente de un familiar o un amigo, lo que hace que esas* noticias falsas*resulten más confiables para el paciente. Esto termina influyendo, porque, como no tenemos una relación familiar con* él*, todo lo que decimos se pone en duda.* (EAP13)

*Porque el paciente llega con una información que cree que es correcta, porque algún conocido se lo dijo. Muchas veces, ni siquiera es un profesional de la salud. Lo dijo,* él (el paciente) *cree que es correcto.* (EH3)

*Hay quienes creen, pero hay otros que no. Lo hacen porque la madre lo dijo, la abuela lo dijo* [...] *vas a administrar el medicamento recetado, y entonces viene el paciente rechazando la administración porque hace daño, porque vio en Google que afecta a algún sistema o porque alguien lo dijo.* (EH7)

El segundo grupo percibido por los profesionales de enfermería estaba formado por personalidades alejadas del círculo personal de los pacientes y de los propios profesionales, pero que, de manera similar al grupo anterior, ejercen una influencia considerable en el proceso de comprensión de la realidad, así como en la construcción de significados sobre la salud de quienes consumen y difunden *fake news*. En este contexto, identificaron a líderes políticos, artistas e *influencers* digitales como elementos de este grupo.

Cabe destacar también la percepción de los participantes sobre la politización de la información en salud, cuyo enfoque puede estar relacionado con el ámbito de intereses de quienes producen y difunden *fake news*, con posibles compromisos de la confianza en las instituciones y los profesionales de la salud.

*En la* (pandemia de) *COVID-19, hubo publicaciones y mensajes de líderes hablando sobre temas de los que no tenían ningún conocimiento.* (TEH3)

*Por ejemplo, últimamente, con respecto a la vacuna, algunas personas difunden información que contradice su eficacia, desanimando a la población a vacunarse. Creo que también está muy relacionado con la política y, con ello, perjudica las políticas de salud.* (EH6)

*E También hay una cuestión política, por ejemplo, del gobierno, difundiendo información que no es cierta para su propio beneficio.* (EH8)

*Recibimos a un paciente que dice que vio un video en YouTube* ^®^ de *tal persona. Muchas veces, la persona que está dando esa información allí ni siquiera es un profesional.* (EAP18)

*Desafortunadamente, tenemos una población con bajo nivel educativo, que no entiende el proceso de salud y enfermedad y cree todo lo que ve en Internet, terminando por difundirlo. Se dejan llevar por personas influyentes que aparecen en los medios. Por ejemplo, artistas o políticos a quienes admiran. Cuando personas en esta posición terminan diciendo algo que desconocen, tienen el papel de influir en personas legas.* (TEH3)

Los profesionales de enfermería reconocen que los operadores de *fake news* establecen tales interacciones de manera significativa en el entorno virtual a partir de las redes sociales. Los significados que los participantes atribuyen a estas plataformas están relacionados con los peligros que ofrecen, especialmente debido a la dinámica que asumen en los procesos de difusión y acceso de *noticias falsas.*

*Internet, Instagram* ^®^ *, que es surrealista, es muy peligroso, y WhatsApp* ^®^ *, que es aún más surrealista.* (EH24)

*Principalmente las redes sociales. Instagram* ^®^ *, Twitter* ^®^ (“X”)*, creo que propagan muchas noticias falsas.* (EH23)

*Últimamente,* las noticias falsas *han cobrado fuerza gracias a WhatsApp* ^®^ *, ¿no?!* (TEAP20);

*Las noticias falsas se están difundiendo* [...] *a través de las redes sociales.* (TEH12)

### Fuentes Confiables: ¿Argumentos de Autoridad o Autoridad de los Argumentos?

Los profesionales de enfermería atribuyen significados que denotan confiabilidad a las fuentes oficiales de instituciones consolidadas, como el Ministerio de Salud, las Secretarías Municipales de Salud y las Normas Técnicas derivadas de esos contextos. Reconocen la confiabilidad de las fuentes periodísticas, sobre todo aquellas que corroboran las fuentes gubernamentales.

El reconocimiento de las fuentes confiables señaladas por los profesionales de enfermería se centra en el argumento de autoridad que representan dichas fuentes. En este sentido, valoran organismos especializados, como la Fundación Oswaldo Cruz y la Agencia Nacional de Vigilancia Sanitaria. Además, los participantes señalaron la importancia de la facilidad de acceso a la información a través de las redes sociales utilizadas por los organismos gubernamentales y especializados.

*Creo que hay que buscar la información de las Normas Técnicas, que son difundidas por la Secretaría de Salud, estar atento a las redes de la municipalidad, participar en las capacitaciones que se ofrecen.* (EAP17)

*Creo que, hoy en* día, *todo es muy fácil. Incluso el propio Ministerio de Salud y la Anvisa tienen Instagram* ^®^ para *desmentir la información que circula en las redes. Así que estos organismos se han incorporado a las redes para educar. Yo trato de informarme a través de ellos.* (EH6)

*El Ministerio de Salud, para mí, es una fuente segura. A veces, cuando se trata de una noticia de salud que salió en los periódicos, ¿no es lo que dicen? Buscar la fuente periodística más segura.* (EH11)

*Creo que son los organismos del gobierno, el Ministerio de Salud, la Fiocruz. Para mí, son ellos quienes cuentan con un grupo de especialistas que estudian y logran transmitir las medidas correctas.* (TEAP20)

Desde una perspectiva descentralizada, los profesionales de enfermería atribuyeron confiabilidad a las fuentes que surgieron de las interacciones en el día a día laboral, tales como jefes/líderes de enfermería y otros enfermeros. Sin embargo, también revelaron cierto grado de escepticismo respecto a la búsqueda de información junto a profesionales que, aunque reconocidos por ellos como referentes de conocimiento, no siempre fueron percibidos, de manera inequívoca, como fuentes seguras de información.

*De alguna manera lo buscamos. Cuando hay algo directamente relacionado con el hospital, le preguntamos al jefe. Al menos, yo pregunto.* (TEH5)

*Considero que los conocimientos del enfermero son fuentes importantes.* (TEAP16)

*Entonces, creo que deben desconfiar, en principio, y luego, siempre escuchar a las personas que son referentes en esa* área*, a quienes estudian el tema. Los enfermeros pueden ser fuentes confiables.* (EH2)

Los participantes también mencionaron como fuentes seguras de información a los medios de comunicación tradicionales, como la radio, la televisión y los periódicos, cuya confiabilidad radica en la autoridad del argumento que los enfermeros y técnicos de enfermería comprenden que se establece a partir del compromiso y la capacidad de investigación de estos medios.

*Buscando en canales de noticias confiables, como los periódicos que ya cuentan con un proceso para verificar la información*. (EH25)

*Medios de información más tradicionales como los periódicos, aunque sean en línea, pero que incluyan el nombre y la referencia de dónde proviene esa información.* (EH14)

*En Internet, uno publica lo que quiere. El periodismo, en la televisión, necesita, de hecho, fundamentos para transmitirte esa información. Además de informar, va al lugar, hace preguntas. Existe un proceso de investigación.* (EH26)

*Siempre busco información en bases de datos y también en noticieros, porque ya cuentan con mecanismos de verificación de la información. Después de la pandemia de COVID-19, creo que se intensificó aún más.* (EAP19)

Para los enfermeros entrevistados, son fuentes confiables de información aquellas relacionadas directamente con la ciencia, a partir de bases de datos especializadas, como LILACS, BDENF, PubMed, además de herramientas más genéricas, como *Google* ^®^. En este contexto, las revistas científicas son fuentes confiables para enfoques específicos, como la discusión de casos.

*La base de datos de salud misma. LILACS, BDENF, PubMed son las que nos dan más respaldo, y no Google* ^®^ *.* (EAP14)

*Creo que el enfermero debe basarse en información de fuentes seguras, como las bases de datos, las bibliotecas virtuales y las revistas científicas.* (EH04)

*Buscar artículos en bases de datos, como LILACS y BDENF, que concentran allí un acervo de revistas científicas.* (EH06)

*También tenemos acceso a los artículos, así que, siempre que hay discusión de casos, buscamos en las revistas científicas y las bases de datos.* (EAP17)

### Conocer Para Intervenir: Elementos de Identificación de Noticias Falsas

Para los participantes del estudio, las *noticias falsas* presentan indicios en su estructura que permiten identificar su naturaleza engañosa. De esta manera, se señalaron algunas acciones, como la necesidad de verificar la fuente de la información y contrastarla con otras fuentes consideradas confiables.

Según lo expuesto, para los profesionales de enfermería, las *noticias falsas* pueden presentar, en el conjunto de sus datos informativos, una parte verdadera que se asocia con una parte falsa. En este sentido, mencionan la autoría, el canal de comunicación en el que se difunde la noticia y el método utilizado como indicadores que garantizan la confiabilidad de la información a la que se accede y se difunde.

*Estas noticias son como teléfono descompuesto. Tienen una mínima parte de verdad, pero quien las va pasando siempre va añadiendo mentiras.* (EH8)

*A veces se cita algo, y esto, en el* ámbito *de la salud, ocurre mucho. La gente ve que se está estudiando algo y ya mezcla los datos de otros estudios, y distorsiona la información.* (EH22)

*Entonces, hay que fijarse en el autor de ese artículo, buscar en más de una fuente. Si es un video, fijarse en la calidad del video, si fue editado.* (EH23)

*Creo que hay que investigar, fijarse en quién fue la persona que escribió esa información. Verificar si es una profesional que realmente sabe sobre ese tema*. (TEH21)

*Además, hay que prestar mucha atención al leer esa información falsa, que normalmente siempre viene con un título sensacionalista, con un nombre conocido que nos deja siempre en la duda.* (EAP15)

*Creo que, en principio, deben desconfiar. Después, siempre hay que escuchar a las personas que son referentes en esa* área*, a quienes estudian el tema.* (EH2)

Sin embargo, los entrevistados, a pesar de señalar estos elementos de identificación, consideraron que las *fake news* son informaciones difíciles de reconocer, como lo demuestran los siguientes fragmentos:

*Cuando vemos las noticias en las redes, es difícil distinguir qué es mentira o verdad en esa información, porque, cada* día *que pasa, están mejor elaboradas.* (EH1)

*A veces, nos surgen dudas cuando nos enfrentamos a esta información falsa. Porque, hoy en* día, *los recursos también están muy avanzados* (TEAP20)

*No voy a decir que sea una tarea fácil. No siempre, a primera vista, logramos identificarlas.* (EAP19)

### Formas de Combatir las Noticias Falsas

Esta subcategoría recoge los datos presentados por los participantes del estudio sobre las acciones que, según ellos, resultan eficaces para combatir la difusión de *noticias falsas* en el ámbito de la salud, incluso en el contexto del trabajo de enfermería, especialmente con los pacientes. A tal fin, los datos revelaron la importancia de la educación en salud como principal estrategia utilizada por los profesionales.

Los recursos señalados por los encuestados presentan diferentes posibilidades para hacer frente a las *noticias falsas*, sobre todo aquellas centradas en la orientación y en recursos visuales, a partir de la elaboración de folletos enfocados en los problemas de salud.

*Creo que la* única *manera es orientar a los pacientes y a sus acompañantes. Es lo que hemos estado haciendo aquí, trayendo a una persona que trabaje en esa* área *para que explique.* (TEH07)

*Creo que los folletos informativos pueden ser una opción. Creo que esta educación sobre la información falsa es del interés de todos.* (EH2)

*Orientación diaria a los pacientes y a los acompañantes. Y creo que es importante que estas acciones de educación involucren a toda la comunidad, a los agentes de salud, al representante comunitario, a las escuelas*. (TEAP16)

*Normalmente he estado hablando y orientando cuando me encuentro con pacientes o familiares que creen en noticias que se difunden falsamente en las redes sociales. No siempre me escuchan, pero cumplo con mi papel de orientar.* (EH1)

*Creo que la educación en salud, que es algo muy presente, incluso en la Atención Primaria, también. Creo que debemos hablar e informar a las personas, llevarles conocimiento. Creo que es una herramienta fundamental, básicamente, para combatir las noticias falsas.* (EAP15)

Otro recurso pedagógico señalado es la necesidad de campañas de concientización con el objetivo de combatir los mitos y tabúes que contribuyen a la propagación y reiteración de las *noticias falsas*.

*Creo que debería haber campañas de concientización sobre el intercambio de esta información, porque esta información acaba teniendo toda esta magnitud porque hay personas que la comparten.* (EH4)

*Creo que se deberían realizar campañas dentro del hospital para concienciar a los pacientes sobre ciertos cuidados. Pienso que, siempre que sea posible, debe haber acciones educativas: pueden ser charlas, rondas de conversación* [...] *creo que debemos acercarnos a ese paciente, tratando de entender sus dudas y aclararlas punto por punto, como hago yo cuando me enfrento a situaciones de falta de información.* (TEH3)

*Una iniciativa interesante que creo que se puede llevar a cabo, igual que se hace con la vacunación, son las campañas de concientización, porque pueden sensibilizar y alertar a las personas de que las* noticias falsas*generan problemas.* (EAP14)

Los enfermeros del ámbito hospitalario mencionaron, como estrategia para hacer frente a las *noticias falsas*, el desarrollo y la promoción de perfiles de verificación de información en las redes sociales, con especial énfasis en *Workplace* ^®^.

*Creo que debería haber una fuente segura, una plataforma, no sé, donde sea posible replicar la información y, al final, que te diga si es verdadera o no.* (EH6)

*Veo que se podría crear algo como Workplace* ^®^ para *el público. Es similar a Facebook* ^®^ *, pero con el objetivo de difundir acciones e información segura dentro de la institución. Me parece una idea interesante que podría utilizarse como estrategia, ya que sería como una red social del hospital y permitiría a los profesionales gestionar la información.* (EH08)

Además, los participantes destacaron la formación continua como una estrategia importante para los profesionales de enfermería, mediante la realización de cursos o charlas, con el objetivo de capacitarlos en conocimientos y actitudes, a fin de trasladar dichos conocimientos a los pacientes.

*Creo que los profesionales necesitamos cursos de capacitación, cursos o charlas que aclaren dudas sobre los temas que suelen aparecer en las noticias falsas.* (EH4)

*Estas capacitaciones son importantes porque nos dan seguridad. Nos hacen reflexionar sobre nuestro* día a día *en el trabajo; aportan nuevos intercambios. Esto hace que nos sintamos más preparados para enfrentar estas noticias cuando el usuario nos las presenta.* (EAP17)

Por lo tanto, del conjunto de principios/subcategorías surgió la teoría sustantiva, basada en la complejidad, que señala la multidimensionalidad para enfrentar las noticias falsas en el ámbito de la salud, desde la perspectiva de la enfermería. El objetivo es reconocer las conexiones entre los operadores de las noticias falsas, las fuentes de información y sus implicaciones simbólicas, entre los argumentos de autoridad y las autoridades del argumento, así como las múltiples formas de combatir las noticias falsas en el ámbito de la salud.

## DISCUSIÓN

El contexto del que surgen las noticias falsas influye tanto o más que la propia información falsa difundida. En este sentido, los líderes políticos y sanitarios deben comprometerse con políticas públicas y estrategias movilizadoras para un conocimiento capaz de romper la lógica basada en la posverdad, mediante inclinaciones limitadas al ámbito de las emociones^(8,11)^. Esta realidad se ve reforzada por burbujas virtuales, impulsadas por tecnologías de comunicación como *WhatsApp* ^®^, *Facebook* ^®^ *, Instagram* ^®^ *, “X”* ^®^ y tantas otras. En conjunto, estos medios estimulan interacciones restringidas a las perspectivas comunes a los grupos de pertenencia virtuales, de modo que se reitera el proceso no dialéctico del conocimiento en grupos restringidos^(4,11,12)^.

Desde la perspectiva de la complejidad^(1)^, la relación de interdependencia entre el grupo y sus integrantes no se establece únicamente por el principio holográfico, en el que el todo contiene las partes y las partes representan el todo. Para el pensamiento complejo, la dinámica de interacción entre estas dimensiones hace emerger, sobre todo, el principio del circuito recursivo, cuyos actores son productores y productos de sí mismos. Así, la reiteración del pensamiento común que reifica las *fake news* se basa en la calidad de las interacciones entre las partes y el todo, cuya relación de confianza ejerce reciprocidad entre ambos, constituyéndose en un factor preponderante en las acciones que motivan el consumo y el intercambio de *fake news* ^(13)^.

La interacción entre los conceptos y principios de esta investigación corrobora el principio del circuito recursivo, al establecer sobre los emisores de noticias falsas un efecto de producto-productor, ya que, al mismo tiempo que emiten noticias falsas, son también consumidores dependientes de ellas^(1)^. En este proceso cíclico, la literatura científica reitera que, en el contexto de la salud, al tratarse de noticias falsas, el discurso de autoridad prevalece sobre la autoridad del discurso^(14)^, razón por la cual las instituciones de salud e investigación, así como los propios enfermeros, deben establecer estrategias que permitan alcanzar proyecciones de valorización de la ciencia^(15)^. Para ello, dichos actores deben ser entendidos como autoridades epistémicas, facilitadoras del acceso a la información para la construcción del conocimiento^(16,17)^, que tiene como objetivo, entre otros, hacer frente a las noticias falsas en la ciencia y la salud^(18)^.

Esta investigación corrobora lo que se ha abordado en la literatura científica sobre la magnitud de las influencias de los actores (líderes e instituciones), que conforman la perspectiva de la autoridad del discurso sobre la difusión y/o la lucha contra las *noticias falsas*. Sin embargo, el nivel de conocimiento científico de las personas que consumen y comparten *noticias falsas* también es un factor determinante en esta realidad^(19)^, lo que reitera la necesidad de mayores inversiones en la formación científica del enfermero^(20)^. En este contexto, se considera que el personal de enfermería, a lo largo de su formación, debe estar preparado para desarrollar competencias de liderazgo, entre las que se encuentra la condición primordial de movilizar esfuerzos hacia un propósito común, bajo una lógica capaz de motivar con el ejemplo, lo que incluye la valorización de la ciencia. Así, se considera que es responsabilidad del enfermero mantenerse informado y actualizado sobre los temas que puedan influir en sus procesos de toma de decisiones, lo que implica la necesidad de vigilancia epistémica y competencia científica para saber interpretar datos y descodificar información que se supone científica^(21)^.

Ante lo expuesto, es posible inferir que la lucha contra la desinformación implica la propia complejidad en la gestión del conocimiento, como la capacidad de consumir, descodificar, comunicar y evaluar la información científica que busca generar conocimientos, cuyas consecuencias pueden reflejarse en comportamientos que afectan la salud. Por lo tanto, también es necesario conocer la dinámica y el origen de la información consumida^(17)^, así como asumir la necesidad de competencia tecnológica y científica para el acceso, el consumo y la difusión de información veraz.

Algunas acciones pueden formar parte de la vigilancia en la lucha contra la desinformación causada por las *fake news*en el ámbito de la salud. Los datos de esta investigación corroboran, además, los indicadores de vigilancia epistémica de la información, ya que, en el periodismo, por ejemplo, la garantía de la integridad de la información recae en el propio contexto periodístico, ya sea por parte del profesional, del medio de difusión o de asociaciones comprometidas con la veracidad de las noticias en la prensa. Sin embargo, otras medidas pueden marcar la diferencia, como la verificación de la fecha de la información difundida^(22)^, ya que el espacio temporal puede hacer que la información obsoleta constituya una *noticia falsa* cuando se difunde como un conjunto de datos actuales. Además, la velocidad con la que se propagan las *noticias falsas* es mayor que la dinámica de las estrategias para combatirlas, entre las que se encuentran las agencias de verificación periodística, que funcionan de acuerdo con la demanda de los usuarios, quienes señalan la necesidad de verificar estas noticias^(23)^.

El uso de recursos tecnológicos en acciones de educación en salud constituye, asimismo, una estrategia importante para combatir la desinformación. No obstante, cabe destacar la importancia de que los profesionales de enfermería busquen estos recursos y brinden información coherente, de calidad y con base científica^(24)^. Para ello, el proceso de comunicación de la ciencia, especialmente en el ámbito de la salud, debe ser una competencia que los profesionales de enfermería desarrollen constantemente^(20)^.

En cuanto a las fuentes, los resultados de esta investigación se ven corroborados por evidencias que señalan la naturaleza de las fuentes de información como un elemento que integra la multidimensionalidad del acceso y la difusión de *fake news* en el ámbito de la salud a través de las redes sociales. En un estudio realizado por la *Iowa State University* con estudiantes universitarios, se demostró que la mayoría (69%) de los participantes logró determinar con éxito la veracidad de las publicaciones sobre salud en las redes sociales. Sin embargo, se destacó que, entre los elementos que orientaron la capacidad de juicio de los estudiantes, se encuentran principalmente la credibilidad otorgada a la fuente, la apariencia y el estilo de diseño de las publicaciones^(19)^.

Desde una perspectiva compleja, la difusión de *fake news* en el ámbito de la salud debe entenderse mediante un análisis de la coyuntura capaz de destacar los matices contextuales que involucran elementos socioeconómicos y culturales^(25)^. Esta interpretación se ve corroborada por una investigación realizada con 5.307 entrevistados en seis países del África subsahariana, cuyos datos pusieron de relieve que, aunque una parte significativa de las noticias falsas sobre salud se difunde accidentalmente, hay una parte considerable de personas que comparten dicha información a propósito. Sin embargo, lo más significativo de estos datos se encuentra en los determinantes individuales, los cuales indicaron que las personas mayores, de sexo masculino y más propensas a asumir riesgos, son los actores más inclinados a compartir información falsa sobre salud^(25)^.

Los datos de esta investigación se ven, por lo tanto, corroborados por la literatura, especialmente en relación con la multidimensionalidad que envuelve el consumo y la difusión de noticias falsas en el ámbito de la salud, a partir de la importancia del contexto, las fuentes y la reiteración de las noticias^(8,16,19,24)^. Sin embargo, se destacan por presentar una perspectiva compleja de la enfermería sobre el tema, especialmente por ser esta la profesión que ocupa el mayor número de puestos de trabajo en el sector de la salud en todos los sistemas del planeta, así como por ser estratégica para la lucha contra la desinformación en el área^(8)^.

Las limitaciones de la investigación están relacionadas con el campo epistémico, ya que la naturaleza de los participantes insertados únicamente en el contexto de la salud pública puede, por ejemplo, conformarse en significados específicos sobre las *fake news* en las áreas de la salud y la enfermería.

## CONCLUSIÓN

Los significados revelados por los profesionales de enfermería tuvieron en cuenta la complejidad de la lucha contra las noticias falsas en la salud y en la propia enfermería, poniendo de manifiesto que la difusión de esta información se produce por múltiples vías y está insertada en dinámicas sociales, políticas y tecnológicas. Para los enfermeros y técnicos de enfermería, independientemente del escenario de actuación, existen desafíos significativos al tratar con pacientes influenciados por fuentes no confiables, ya sean familiares, redes sociales o figuras públicas. Además, los resultados de esta investigación alcanzaron el objetivo del estudio al señalar la importancia del desarrollo de mecanismos institucionales de verificación y de la promoción de campañas educativas como estrategias esenciales para minimizar los impactos de las noticias falsas en la atención de la salud.

## DISPONIBILIDAD DE DATOS

Todo el conjunto de datos que apoya los resultados de este estudio fue publicado en el propio artículo.
